# Exploring Ca^2+^ Dynamics in Myelinating Oligodendrocytes through rAAV-Mediated jGCaMP8s Expression in Developing Spinal Cord Organ Cultures

**DOI:** 10.1523/ENEURO.0540-23.2024

**Published:** 2024-06-03

**Authors:** Maria Pachetti, Anabela Palandri, Fernanda de Castro Reis, Luca Recupero, Laura Ballerini

**Affiliations:** Scuola Internazionale Superiore di Studi Avanzati, Trieste 34146, Italy

**Keywords:** adeno-associated virus, calcium, live imaging, myelinating oligodendrocytes, spinal cord organ cultures

## Abstract

Oligodendrocytes, the myelin-producing glial cells of the central nervous system (CNS), crucially contribute to myelination and circuit function. An increasing amount of evidence suggests that intracellular calcium (Ca^2+^) dynamics in oligodendrocytes mediates activity-dependent and activity-independent myelination. Unraveling how myelinating oligodendrocytes orchestrate and integrate Ca^2+^ signals, particularly in relation to axonal firing, is crucial for gaining insights into their role in the CNS development and function, both in health and disease. In this framework, we used the recombinant adeno-associated virus/Olig001 capsid variant to express the genetically encoded Ca^2+^ indicator jGCaMP8s, under the control of the myelin basic protein promoter. In our study, this tool exhibits excellent tropism and selectivity for myelinating and mature oligodendrocytes, and it allows monitoring Ca^2+^ activity in myelin-forming cells, both in isolated primary cultures and organotypic spinal cord explants. By live imaging of myelin Ca^2+^ events in oligodendrocytes within organ cultures, we observed a rapid decline in the amplitude and duration of Ca^2+^ events across different in vitro developmental stages. Active myelin sheath remodeling and growth are modulated at the level of myelin–axon interface through Ca^2+^ signaling, and, during early myelination in organ cultures, this phase is finely tuned by the firing of axon action potentials. In the later stages of myelination, Ca^2+^ events in mature oligodendrocytes no longer display such a modulation, underscoring the involvement of complex Ca^2+^ signaling in CNS myelination.

## Significance Statement

Determining the sources and mechanisms driving Ca^2+^ events in mature oligodendrocytes, typically studied through restricted transgenic lines, has proven to be challenging. To address this, we employed the recombinant adeno-associated virus/Olig001 to selectively express jGCaMP8s, under the transcriptional control of the myelin basic protein promoter, to monitor Ca^2+^ activity specifically in myelinating and mature oligodendrocytes in vitro and ex vivo. Our findings indicate that Ca^2+^ dynamics undergoes maturation-dependent modulation and that neuronal activity can have a different impact on Ca^2+^ activity across developmental stages. Our research introduces a valuable genetic tool for monitoring Ca^2+^ signaling in myelin-forming cells to investigate how Ca^2+^ regulation affects oligodendrocyte function and dynamic interactions with axons.

## Introduction

Glial cells are key modulators of neural network formation and function (Allen and Lyons, 2018). Among these, oligodendrocytes, the CNS myelinating glia, extend segments of the lipid-rich myelin membrane in between nodes of Ranvier on numerous axons, facilitating rapid saltatory action potential conduction. Myelinating cells are adaptable and dynamically regulate the number, distribution, length, and thickness of myelin sheaths in response to neuronal activity (Paez and Lyons, 2020). Myelinating oligodendrocytes play a crucial function in providing metabolic support to axonal processes, and altered or dysfunctional axon–glia coupling can lead to neuronal/axonal degeneration (Monje, 2018). Dynamic interactions between myelinating oligodendrocytes and axons include ion homeostasis, regulated by channels localized in the myelin sheath at the axon–myelin interface. As a ubiquitous signaling molecule and second messenger, the dynamic of intracellular Ca^2+^ concentration ([Ca^2+^]_i_) in oligodendrocytes can directly influence the formation and remodeling of myelin and may underlie other, yet unknown, cellular functions (Giorgi et al., 2018; Paez and Lyons, 2020).

During development, the modulation of [Ca^2+^]_i_ affects various aspects of oligodendrocyte progenitor cell (OPC) maturation (Wake et al., 2011), including their differentiation and the myelin sheath formation and growth (Baraban et al., 2018; Krasnow et al., 2018; Battefeld et al., 2019). On the other hand, demyelination occurs in the immune-mediated disease multiple sclerosis, and it is commonly seen in traumatic brain injury, spinal cord injury, stroke, and aging (Chen et al., 2020). Activation of Ca^2+^ channels and receptors in OPCs and oligodendrocytes by neurotransmitters converges to regulate [Ca^2+^]_i_, making Ca^2+^ a signaling crossroad and a central candidate mediator tuning myelination (Paez and Lyons, 2020). Transgenic animal models, ad hoc modified to express genetically encoded Ca^2+^ indicators (GECIs), are conventionally used for studying Ca^2+^ signaling in mammalian oligodendrocytes (Lu et al., 2023). Although these models have provided valuable insights, they have substantial limitations, including costly breeding schemes and financial and animal welfare concerns, and are generally limited to one gene target at a time. Furthermore, relying on transgenic GECI-expressing lines restricts the flexibility to adopt new generations of GECIs with enhanced performance (Grødem et al., 2023).

This study introduces a recombinant adeno-associated virus (rAAV) to achieve robust expression of targeted GECIs in myelinating oligodendrocytes. By integrating the recently developed jGCaMP8s, with enhanced sensitivity and kinetics (Zhang et al., 2023), with the chimeric AAV/Olig001 capsid variant, which exhibits a preferential tropism for oligodendrocytes in vivo (Powell et al., 2016), our AAV-pMBP-jGCaMP8s-WPRE viral vector emerges as a potent and innovative tool. We show the vector efficiency in reporting live imaging of intracellular Ca^2+^ dynamics specifically and selectively in myelinating oligodendrocytes, with sustained expression persisting for >2 weeks in spinal cord organotypic slices. Leveraging our knowledge of the molecular regulators of Ca^2+^ in other cells, including neurons and astrocytes, we are now beginning to understand the factors converging on the regulation of Ca^2+^ in oligodendrocytes during distinct stages of myelination. The ability to localize jGCaMP8s in myelinating oligodendrocytes provides the possibility to uncover the nature of Ca^2+^ signaling within and between cells.

## Materials and Methods

### Mice husbandry

All animal studies were carried out with approval of the European Union (EU) and Italian Ministry of Health according to its regulations, under guidelines 2010/63/UE and Decree 26/14. The project was approved by SISSA Animal Care and Use Committee. Animal use was approved by the Italian Ministry of Health 22DABN1WO and 22DABNYQA, in agreement with the EU Recommendation 2007/526/CE.

### Organotypic spinal cord cultures

Organotypic spinal cord and dorsal root ganglia slices were obtained from C57BL/6J mouse embryos at Embryonic Day 12.5. Pregnant mice were exposed to a CO_2_ overdose and decapitated, and fetuses were delivered by cesarean section. Isolated fetuses were decapitated, and their backs were isolated from low-thoracic and high-lumbar regions and transversely sliced with a tissue chopper. Slices (275 μm) were cleaned by dissection, fixed on a glass coverslip with fibrin glue, that is, reconstituted chicken plasma (Rockland Immunochemicals) clotted with thrombin (Merck Millipore) and cultured within flat-bottom 15 ml screw cap tubes containing 1 ml standard Dulbecco's modified Eagle's medium (DMEM; Thermo Fisher Scientific) supplemented with 25% fetal bovine serum (FBS; Thermo Fisher Scientific), B27 (Thermo Fisher Scientific), 50 units/ml penicillin–streptomycin (Invitrogen), and mouse nerve growth factor (10 ng/ml, Alomone Labs). The tubes were placed in a roller drum, which rotated at a rate of 120 rph, at 37°C with 5% CO_2_. Experiments were performed on Day In Vitro 14 (DIV14) and DIV21.

### OPC isolation and oligodendrocytes cultures

OPC cultures were generated from 0- to 3-d-old Wistar rat pups using a modified protocol from Dai et al. (2014). The mixture was placed into poly-L-ornithine-coated 75 cm^2^ flasks and grown in DMEM high glucose (Thermo Fisher Scientific) with 10% FBS for 10–12 d. OPCs were purified from mixed glial cells by a shake-off procedure. Microglia were removed by shaking (100 rpm, 1 h, 37°C); the medium was replaced, and OPCs were purified by shaking overnight (ON; 210 rpm, 37°C). The medium containing cells was collected and filtered (pore size, 70 μm) into noncoated Petri dishes for attachment of astrocytes (1 h). Then, OPCs were centrifuged at 1,200 × *g* for 5 min, and cells were resuspended in a medium containing DMEM, 100× oligodendrocyte supplement (DMEM, 10.2 mg/ml BSA, 6 μg/ml progesterone, 1.61 mg/ml putrescine, 500 ng/ml sodium selenite, 2.5 μg/ml insulin, 200 mM L-glutamine, 50 μg/ml holo-transferrin, 1× N2; Thermo Fisher Scientific) plus 10 ng/ml PDGF and FGF. Cells were cultured in a humidified incubator with 5% CO_2_, with medium changed every 2 d. To initiate differentiation, we shifted the cells to mitogen-free N2 media plus 15 nM triiodothyronine (T3, Sigma-Aldrich). For calcium imaging assays, 1 × 10^5^ cells/coverslip were plated in poly-D-lysine-coated coverslips (12 × 24 mm).

### Immunofluorescence and confocal microscopy

Cells and spinal cord tissues grown on glass coverslips were washed twice with phosphate-buffered saline (PBS) and fixed with 4% (*w*/*v*) paraformaldehyde (Sigma-Aldrich), in PBS for 10 or 30 min at room temperature (RT), respectively. After three washes with PBS, the cells/tissues were incubated for 1 h at RT with a 5% (*v*/*v*) solution of goat serum in PBS permeabilized with 0.3% (*w*/*v*) Triton X-100 (TX110; Sigma-Aldrich). Samples were incubated with primary antibodies ON in a 5% (*v*/*v*) solution of goat serum in PBS with 0.3% (*w*/*v*) Triton X-100. After three washes with PBS, cells/tissues were incubated for 2 h at RT with secondary antibodies. Following three washes with PBS, cell nuclei were stained with 4′,6-diamidino-2-phenylindole (DAPI) dye (Invitrogen). The coverslips were mounted using Fluoromount-G Mounting Medium (Invitrogen). Confocal images were acquired using a Nikon A1R confocal microscope (Nikon), equipped with 4× (NA 0.20), 20× (NA 0.75), and 40× (NA 0.95) air objectives and 60× (1.35 NA) oil immersion objective. Images (1,024 × 1,024 pixels) were processed using the Fiji ImageJ software. The final images were compiled using the Adobe Photoshop CC software. The following primary antibodies were used: rabbit anti-IbaI (1:500, MA5-36257, Invitrogen); mouse anti-GFAP (1:1000, G3893, Sigma-Aldrich), rabbit anti-myelin basic protein (MBP; 1:1000, ab40390, Abcam), and mouse anti-SMI-32 (1:1000, 801701, BioLegend). Secondary antibodies used were as follows: Alexa Fluor 633 goat anti-mouse, Alexa Fluor 633 goat anti-rabbit, Alexa Fluor 594 goat anti-rabbit, and Alexa Fluor 594 goat anti-mouse (1:1000, Invitrogen). Propidium iodide (PI; Sigma-Aldrich) was used at 5 μg/ml in the culture medium to label dead cells in spinal cord organotypic slice cultures.

### Plasmid construct

A plasmid containing the jGCaMP8s and the woodchuck hepatitis virus posttranscriptional regulatory element (WPRE) flanked by AAV2 inverted terminal repeats was used as a recipient plasmid (Addgene). It was digested by ApaI and BamHI-HF to replace the human synapsin promoter with the 1.9 kb MBP gene promoter, amplified by PCR from the plasmid (Addgene; FW, atgctctaggaagatctctgcagagggcccgagctccttcctgcttaggc; RV, tgatgatggtgatgatgcatggtggcgggtggatcctattcgagcttccggaagctg; Sigma-Aldrich; Table 1). To generate AAV-MBP-jGCaMP8s-WPRE, we performed a Gibson Assembly reaction by combining the digested backbone with the amplified MBP promoter (insert, backbone; ratio, 3:1 *w*/*w*). The resulting vector was validated by sequencing (detailed data reported in Extended Data Table 1-1).

**Table 1.** A comprehensive list of plasmids, primers, and probes used in this work

Vector name	Addgene no.
pGP-AAV-syn-jGCaMP8s-WPRE	162374
AAV_pMBP-EGFP-caax	190155
pAdDeltaF6	112867
Olig001	170716
Primers and Probes	Sequence
MBP (FW)	5′-CAGAGACACGGGCATCCTTG-3′
MBP (RV)	5′-GTGTGTGAGTCCTTGCCAGAG-3′
MBP probe	5′-TxRd-TCCATCGGGCGCTTCTTTAGCGGTGACA-BHQ2-3′
GAPDH (FW)	5′-TTCAAGTGGGCCCCGG-3′
GAPDH (RV)	5′-GTGAGGCCGGTGCTGAGTAT-3′
GAPDH probe	5′-Cy5.5-ATGGTGGTGAAGACACCAGTAGACTCCACG-BHQ3-3′

10.1523/ENEURO.0540-23.2024.t1-1 Table 1-1 Download Table 1-1, XLS file.

### Virus production

The AAV production protocol was adapted from Chakrabarti et al. (2020). Briefly, HEK293T cells, obtained from American type Culture Collection, were cultured in DMEM (Thermo Fisher Scientific) supplemented with 10% FBS and 100 units/ml penicillin–streptomycin (Invitrogen) using a two-culture chambers CellSTACK (Corning). At each passage, cells were trypsinized for 1–3 min with 0.25% trypsin and 1 mM EDTA in PBS, pH 7.4 at 37°C, plated, and maintained for 2 d before transfection. Then, cells were triple transfected with the following plasmids: AAV-MBP-jGCaMP8s-WPRE (cargo) and helper and Rep/Cap Olig001 (Addgene) in a 1:2:1 (*w*/*w*) ratio using a linear polyethylenimine (PEI) reagent (Polysciences), at 0.33 mg/ml (1:11 plasmid:PEI *w*/*v*; Table 1). Three days after transfection, cells and media were harvested and treated with Triton X-100 and RNase A (Sigma-Aldrich) to a final concentration of 0.5% and 5 μg/ml, respectively, shaking for 1 h at 37°C. This mixture was centrifuged at 3,700 × *g* at RT for 30 min to remove debris and then concentrated by tangential flow filtration using the Vivaflow 200 cassette 100 kDa molecular weight cutoff (MWCO; Sartorius).

The concentrated medium containing rAAV particles was purified through an iodixanol column (OptiPrep density gradient medium; Serumwerk Bernburg) by ultracentrifugation (Optima XE-90 Ultracentrifuge; Beckman Coulter) at 44,400 rpm for 2 h at 18°C. After collecting the 40% layer, rAAV particles were further concentrated using a 50 kDa MWCO filter (Amicon, ultracentrifugal filters), followed by buffer exchange to 0.001% PBS–pluronic acid 200 mM NaCl solution. Viral titers (vg/ml) were measured by probing for inverted terminal repeats (FW, GGAACCCCTAGTGATGGAGTT; RV, CGGCCTCAGTGAGCGA) with quantitative polymerase chain reaction (qPCR) using a SsoAdvanced Universal SYBR Green Supermix (Bio-Rad Laboratories), according to the manufacturer's instructions. The protocol was optimized to produce rAAVs at high titer [⋍5 × 10^14^ vector genomes (vg/ml)]. Due to the extensive virus's nomenclature, both the following text and images designate it as AAV-mbp:jGCaMP8s.

### Quantification of AAV:mbp-jGCaMP8s transduction efficiency

The quantification of the viral expression efficiency was assessed using the “Analyze particles” function in the Fiji ImageJ software, employing *Z*-stacks of 20× or 40× images obtained from both the oligodendrocyte dissociated culture and the ventral horn of the spinal organ cultures at DIV14 and DIV21. This function identifies colocalization between eGFP+ cells and MBP+ cells, targeting transduced myelinating oligodendrocytes.

### Live calcium imaging

For in vitro live calcium imaging, Oregon Green 488 BAPTA-1 AM (Invitrogen) and AAV-mbp:jGCaMP8s were employed. Primary cultures of oligodendrocytes were incubated with 4 µM Oregon Green 488 BAPTA-1 AM for 40 min (37°C; 5% CO_2_; Rauti et al., 2020) or infected at DIV4 (at a final concentration of 2.3 × 10^8^ v*g* in 2.2 ml) and recorded at DIV8. The samples were then placed in a coupled device camera and preincubated at 37°C in an extracellular solution for 15 min prior to live Ca^2+^ imaging. Then, the samples were mounted on an inverted microscope (Nikon Eclipse Ti-U) and continuously perfused at RT with the same extracellular solution containing the following (in mM): 150 NaCl, 4 KCl, 1 MgCl_2_, 2 CaCl_2_, 10 HEPES, 10 glucose; pH 7.4. Cells were visualized with a 40× objective (Plan Fluor 0.6 × NA), and recordings were performed from visual fields (680 × 680 µm^2^, binning 4 × 4). Samples were excited with a mercury lamp at 488 nm. We used a 395 nm dichroic mirror and DN filter (1/32); images were acquired by an ORCA-Flash 4.0 V2 sCMOS camera (Hamamatsu) and a setup controlled by the HCImage Live software (Hamamatsu). The exposure time ranged from 500 ms to 2 s to prevent bleaching. Live imaging recordings last 30 min for each sample, with movies captured every 10 min.

For ex vivo live Ca^2+^ imaging, AAV-mbp:jGCaMP8s was utilized with the same technical settings employed for in vitro recording. Organotypic spinal cord cultures were infected at DIV7 or DIV14 and recorded after 7 d. The samples were continuously perfused at RT in an extracellular solution with the following composition (in mM): 152 NaCl, 4 KCl, 1 MgCl_2_, 2 CaCl_2_, 10 HEPES, 10 glucose; pH 7.4. Samples were recorded for a total of 30 min, with movies collected every 10 min. Tetrodotoxin (TTX, HB1035, Hello Bio) was added into the extracellular solution, reaching a final concentration of 1 µM. Following a 20 min perfusion at RT, samples were recorded for a total of 30 min, with movies collected every 10 min. Additionally, we conducted an ex vivo longitudinal study, recording Ca^2+^ signaling in oligodendrocytes at DIV14, DIV16, and DIV21 within the same field of view. Organotypic spinal cord slices were infected at DIV7 for 7 d. Live imaging recordings in extracellular solution last 20 min for each sample, with movies captured every 10 min. Postrecording at DIV14, half of the medium containing AAV-mbp:jGCaMP8s was replaced with fresh medium, while after the recording at DIV16, 25% of the medium was replaced with a fresh medium. Following the recording at DIV21, the samples were treated for 20 min with PI and fixed. In the context of this study, we define “Ca^2+^ events” as Ca^2+^ waves, specifically referring to events lasting longer than 1 s.

### Electrophysiology and calcium imaging of oligodendrocytes

Live Ca^2+^ imaging of oligodendrocytes and single-cell electrophysiology recordings from spinal neurons were simultaneously conducted in the ventral area of DIV14 organotypic cultures before and after TTX (1 μM) application. Briefly, whole-cell patch–clamp recordings were performed from ventral interneurons visually identified through a microscope (Nikon) on the basis of previously reported criteria (Avossa et al., 2003). During experiments, slices were perfused with extracellular solution as follows (in mM): 152 NaCl, 4 KCl, 1 MgCl_2_, 2 CaCl_2_, 10 HEPES, 10 glucose, pH 7.4.

Patch pipettes (4–6 MΩ) contained the following (in mM): 120 K gluconate, 20 KCl, 10 HEPES, 10 EGTA, 2 MgCl_2_, 2 Na_2_ATP. The pH was adjusted to 7.3 with KOH (295 mOsm). All recordings were performed at RT. Data was recorded via the NIS-Elements D 4.66 acquisition software with a 40×/0.80w Nikon Fluor objective. Electrophysiological responses were amplified via MultiClamp 700B (Axon CNS Molecular Devices), with the amplifier's gain set at 1, membrane current 0.5 V/nA, sampled, and digitized at 2 kHz via DigiData 1550B and pCLAMP software (Axon Instruments) for off-line analysis.

In voltage-clamp mode, we considered only recordings where uncompensated series resistance values were <20 MΩ, enabling recordings of synaptic currents without significant distortion. Spontaneous postsynaptic currents were monitored at a holding potential of −56 mV, not compensated for liquid junction potential (−14 mV). Ca^2+^ events (defined as above) were recorded with an exposure time of 150 ms and 2 × 2 binning.

### Ca^2+^ live imaging analysis

Recorded movies were analyzed off-line using the Fiji ImageJ and Clampfit (pClamp suite, 10.7 version; Molecular Devices) software. [Ca^2+^]_i_ waves were expressed as a fractional amplitude increase Δ*F* / *F*_0_, where *F*_0_ is the baseline fluorescence level and Δ*F* is the rise over the baseline. *F*_0_ was determined by calculating the average fluorescence intensity during the period of cellular inactivity (for Oregon Green) or as the average of the lowest recorded fluorescence intensity within the same field of view and in the same movie (for AAV-mbp:jGCaMP8s). We defined Ca^2+^ waves as increases in Δ*F* / *F*_0_ exceeding 20% of the average maximum peak of amplitude recorded in each movie. We defined the frequency of Ca^2+^ waves as the number of waves observed throughout each recording. We defined as duration the temporal difference between the endpoint and the starting point of the recorded wave; we defined amplitude as the fluorescence intensity at the peak of the detected wave. Data pooled from different cellular regions were integrated to generate a single data point presented as average frequency, average duration, and average amplitude. The collected data were derived from ≥5 experiments, each employing a different culture.

### RNA extraction and RT-PCR

Total mRNA was isolated from the spinal cord organotypic cultures at DIV14 and DIV21 using a standard TRIzol RNA extraction protocol. The RNA obtained (50 ng) was reverse-transcribed and amplified using Luna Universal Probe One-Step RT-qPCR Kit (New England Biolabs) with sequence-specific primers (1 μM) and probes (200 nM) in a 20 μl reaction, following the manufacturer's instructions. Primers and probes sequences are listed in Table 1. Quantification of *Mbp* gene expression was performed, and the expression of the housekeeping gene *Gapdh* was used for normalization. Amplifications were performed using a Bio-Rad Laboratories CFX96 machine. The real-time qPCR program was executed in accordance with the manufacturer's instructions (annealing 60°C for 30 s).

### Polyacrylamide gel electrophoresis (SDS-PAGE) and Western blotting

Spinal cord organotypic culture lysates were prepared in the RIPA buffer (50 mM Tris–HCl, pH 7.5, 150 mM NaCl, 1% (*v*/*v*) Nonidet P-40, 0.2% (*p*/*v*) sodium deoxycholate) supplemented with a cocktail of protease inhibitors (Roche Diagnostics). For the quantification of the total protein in cellular lysates, the Bradford assay (Bio-Rad Laboratories) was used. All samples were diluted with the Laemmli buffer in the presence of 5% (*v*/*v*) β-mercaptoethanol (reduced SDS-PAGE) and heated at 95°C for 5 min. Proteins were resolved by SDS-PAGE and transferred onto a polyvinylidene fluoride membrane (Sigma-Aldrich). Transferred protein bands were visualized by staining with 0.2% (*p*/*v*) Ponceau S in 1% (*v*/*v*) acetic acid. Subsequently, for MBP (1:1000, Abcam) and MAG (1:1000, D4G3 Cell Signaling Technology) detection, membranes were blocked for 1 h at RT with 5% (*p*/*v*) BSA in PBS containing 0.1% Tween 20 (PBS-T). For the detection of Gapdh (1:1000, 60004-1-lg, Proteintech), membranes were blocked with 5% (*p*/*v*) nonfat dry milk in PBS. Membranes were then incubated ON at 4°C with the primary antibody diluted in the appropriate blocking solution, washed three times with PBS-T or TBS-T, and incubated for 1 h at RT with the secondary antibody diluted in the blocking solution. Protein bands were visualized using the chemiluminescence detection system (Uvitec). Densitometric analysis of immunoblots was performed using the Fiji ImageJ software.

### Statistics

All experiments were performed as independent biological replicates. Statistical analyses and graphs were generated using GraphPad Prism 8.0.1. Testing was performed for normality by the D’Agostino–Pearson omnibus normality test. For normally distributed data, two-tailed unpaired parametric Student's *t* tests were utilized, with equal variance confirmed by the *F* test. Nonnormally distributed data were analyzed using a two-tailed Mann–Whitney test. Pearson's correlation test was used to assess correlation. Throughout all analyses, *p < 0.05, **p < 0.01, ***p < 0.001, and ****p < 0.0001. Detailed statistical methods for each figure are provided in an attached table of statistics (Table 2). The number of cultures analyzed for each experiment and each condition was ≥3, unless otherwise stated.

**Table 2.** Summary statistics

Figure	Parameter	Type of test	Sample size	Statistical data
Figure 2*H*	Correlation amplitude versus duration of Ca^2+^ events	Pearson’s correlation *t* test, two-tailed	*N* = 315 oligodendrocyte processes	*r* = 0.67 ***p *< 0.0001**
Figure 2*J*	Frequency, duration and amplitude of Ca^2+^ events	Unpaired parametric *t* test, two-tailed	*N* = 10 (jGCamP8s), *N* = 15 (Oregon Green) movies	F: *t* = 0.8814; df = 23; *p *= 0.386 D: *t* = 0.3840; df = 27; *p *= 0.704 A: *t* = 0.6385; df = 29; *p *= 0.528
Figure 3*E*	Efficiency (%)		*N* = 3 (eGFP), *N* = 3 (eGFP/MBP)	eGFP (mean ± SEM): 89.29 ± 8 eGFP/MBP (mean ± SEM): 74.91 ± 5.9
Figure 3*I*	Average frequency of Ca^2+^ events	Unpaired parametric *t* test, two-tailed	220 (control),135 (TTX) oligodendrocyte processes; *N* = 16 (control), *N* = 10 (TTX) movies	*t* = 0.3554; df = 24; *p *= 0.7254
Figure 3*J*	Average duration of Ca^2+^ events	Unpaired *t* test Two-tailed Mann–Whitney	220 (control),135 (TTX) oligodendrocyte processes; *N* = 16 (control), *N* = 10 (TTX) movies	***p *= 0.0328**
Figure 3*K*	Average amplitude of Ca^2+^ events	Unpaired *t* test Two-tailed Mann–Whitney	220 (control), 135 (TTX) oligodendrocyte processes; *N* = 16 (control), *N* = 10 (TTX) movies	***p *< 0.0001**
Figure 4*C*	Average frequency of Ca^2+^ events	Unpaired *t* test Two-tailed Mann–Whitney	183 (control), 49 (TTX) oligodendrocyte processes; *N* = 13 (control), *N* = 5 (TTX) movies	*t* = 0.3735; df = 16; *p *= 0.9241
Figure 4*D*	Average duration of Ca^2+^ events	Unpaired *t* test Two-tailed Mann–Whitney	183 (control), 49 (TTX) oligodendrocyte processes; *N* = 13 (control), *N* = 5 (TTX) movies	*p *= 0.2751
Figure 4*E*	Average amplitude of Ca^2+^ events	Unpaired *t* test Two-tailed Mann–Whitney	183 (control), 49 (TTX) oligodendrocyte processes; *N* = 13 (control), *N* = 5 (TTX) movies	*p *= 0.3140
Figure 4*G*	Correlation amplitude vs duration of Ca^2+^ events	Pearson’s correlation Two-tailed	*N* = 183 (control), *N* = 49 (TTX) oligodendrocyte processes	*r* = 0.46, ***p *< 0.0001** (control); *r* = 0.22, ***p *= 0.005** (TTX)
Figure 4*H*	Average frequency of Ca^2+^ events	Unpaired parametric *t* test, Two-tailed	220 (DIV14), 183 (DIV21) oligodendrocyte processes; *N* = 16 (control), *N* = 13 (TTX) movies	*t* = 1.089; df = 27; *p *= 0.2857
Figure 4*I*	Average duration of Ca^2+^ events	Unpaired *t* test Two-tailed Mann–Whitney	220 (DIV14), 183 (DIV21) oligodendrocyte processes; *N* = 16 (control), *N* = 13 (TTX) movies	***p *< 0.0001**
Figure 4*J*	Average amplitude of Ca^2+^ events	Unpaired *t* test Two-tailed Mann–Whitney	220 (DIV14), 183 (DIV21) oligodendrocyte processes; *N* = 16 (control), *N* = 13 (TTX) movies	***p *< 0.0001**
Figure 4*L*	Number of branches per OL	Unpaired parametric *t* test Two-tailed	11 (DIV14), 11 (DIV21) oligodendrocyte processes; *N* = 3 (DIV14), *N* = 3 (DIV21) movies	*t* = 2.371; df = 20, ***p *= 0.0279**
Figure 4*M*	Sheath length	Unpaired parametric *t* test Two-tailed	11 (DIV14), 11 (DIV21) oligodendrocyte processes; *N* = 3 (DIV14), *N* = 3 (DIV21) movies	*t* = 0.1436; df = 18, *p *= 0.8874
Figure 4*N*	Total myelin sheath per OL	Unpaired *t* test Two-tailed Mann–Whitney	11 (DIV14), 11 (DIV21) oligodendrocyte processes; *N* = 3 (DIV14), *N* = 3 (DIV21) movies	***p *= 0.0433**
Figure 4*O*	MBP and MAG protein expression	Unpaired parametric *t* test Two-tailed	*N* = 3 (DIV14), *N* = 3 (DIV21) spinal cord organotypic cultures	MBP: *t* = 3.379; df = 4, ***p *= 0.0278** MAG: *t* = 3.682; df = 4, ***p *= 0.0212**
Figure 4*P*	MBP RNA expression	Unpaired *t* test Two-tailed Mann–Whitney	*N* = 5 (DIV14), *N* = 5 (DIV21) spinal cord organotypic cultures	***p *= 0.0317**

Bold values are statistically significant, which means *p* < 0.05. Figure 2H: ****p < 0.0001; Figure 3J: *p = 0.0328; Figure 3K: ****p < 0.0001; Figure 4G: ****p < 0.0001, **p = 0.005; Figure 4I: ****p < 0.0001; Figure 4J: ****p < 0.0001; Figure 4L: *p = 0.0279; Figure 4N: *p = 0.0433; Figure 4O: *p = 0.0278, *p = 0.0212; Figure 4P: *p = 0.0317.

## Results

**Figure 1.**
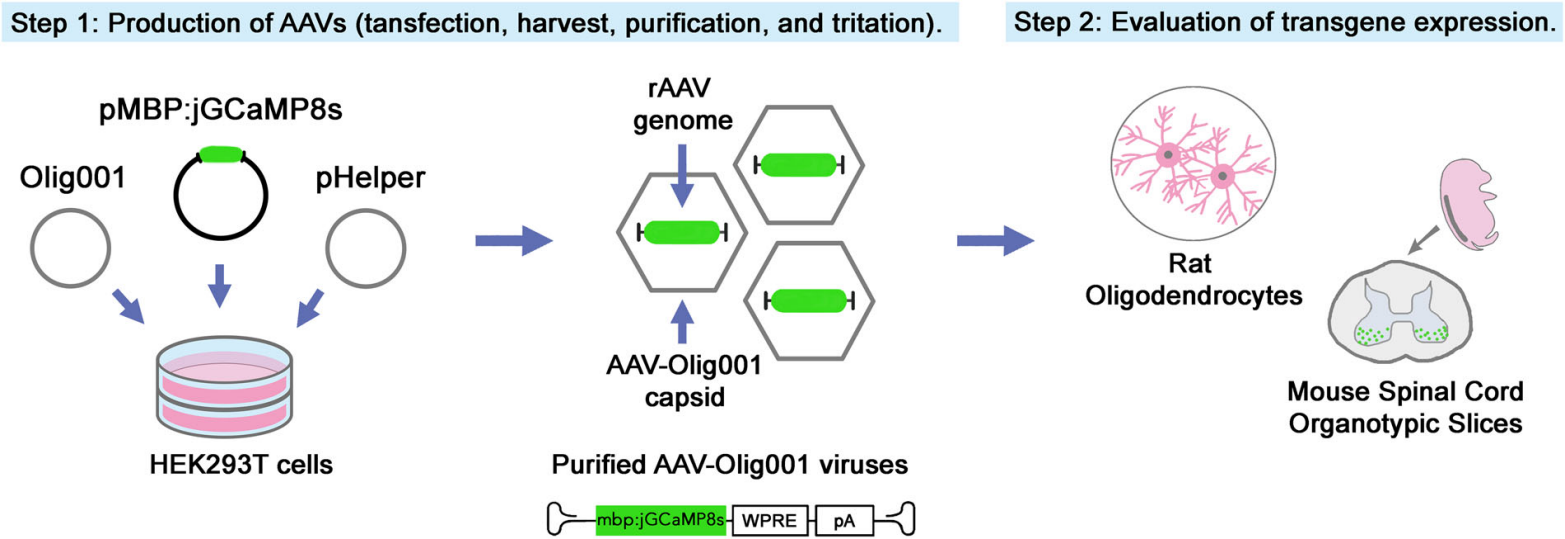
Protocol overview. The procedure consists of two main steps: AAV production and evaluation of transgene expression through Ca^2+^ imaging analysis in primary oligodendrocyte and in organotypic spinal cord cultures. The AAV-mbp:jGCaMP8s is obtained through a triple-plasmid transfection process using HEK293T cells, including the cloned vector containing a green fluorescent reporter (jGCaMP8s), the Rep/Cap plasmid Olig001, and the helper plasmid (Step 1). After collection, purification, and titration, AAV-mbp:jGCaMP8s viral particles were administered to primary oligodendrocyte cultures or organotypic spinal cord slices (Step 2).

We engineered a rAAV to deliver the newly released GECI transgene, specifically jGCaMP8s, under the transcriptional control of the MBP promoter to drive its expression specifically in myelinating oligodendrocytes. Purified viral particles were used to infect both primary rat oligodendrocyte cultures and mouse organotypic spinal cord slices (Fig. 1).

### AAV-mbp:jGCaMP8s expression in oligodendrocytes in vitro

**Figure 2.**
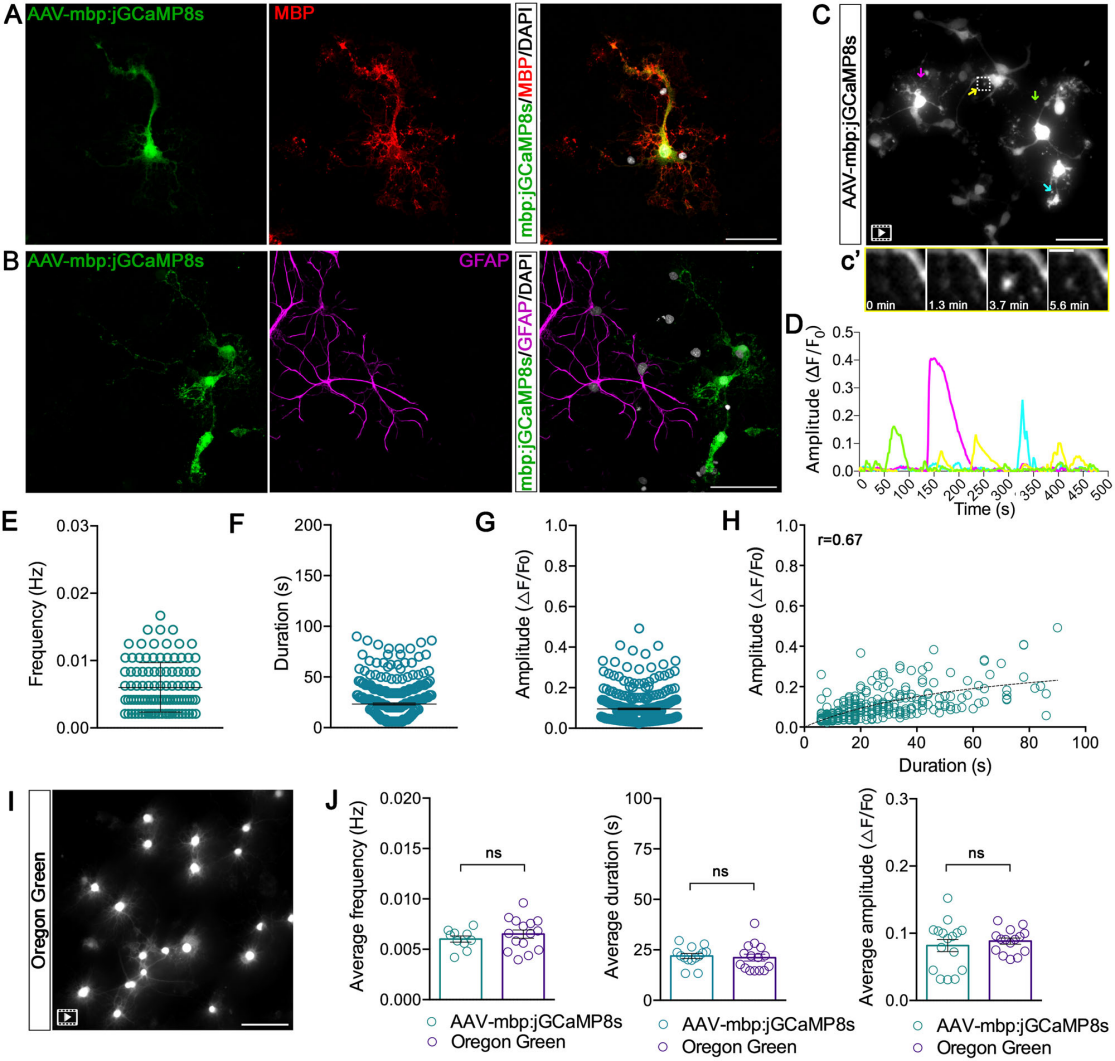
Live imaging reveals Ca^2+^ activity in primary oligodendrocyte cultures using AAV-mbp:jGCaMP8s. ***A***, Representative confocal micrograph of myelinating oligodendrocytes (DIV8) showing eGFP expression for jGCaMP8s (green) and mature oligodendrocytes (MBP, red). Nuclei (white) were labeled with DAPI dye. Scale bar, 100 μm. See Extended Data Figure 2-1 for more details. ***B***, Representative confocal micrograph of myelinating oligodendrocytes showing eGFP expression for jGCaMP8s (green) and of GFAP^+^ astrocytes (magenta). Nuclei (white) were labeled with DAPI dye. Scale bar, 100 μm. ***C***, Maximum intensity projection of a 3D *z*-stack, illustrating an extended-duration movie highlighting jGCaMP8s-expressing myelinating oligodendrocytes. Arrowheads indicate oligodendrocyte processes shown in corresponding colors in fluorescent traces in ***D***. Scale bar, 100 μm. Frames from the time-lapse imaging are indicated with a white box corresponding to panel ***c*’** (Movies 1 and 2). Scale bar, 10 μm. ***D***, Representative traces of Δ*F* / *F*_0_ over time. ***E***, Distribution of Ca^2+^ events frequencies (Hz) per oligodendrocyte process. ***F***, Distribution of Ca^2+^ events duration per oligodendrocyte process. ***G***, Distribution of Ca^2+^ events amplitude per oligodendrocyte process. ***H***, Correlation between amplitude and duration per individual Ca^2+^ events (Pearson's correlation test, *r* = 0.67; *p* < 0.0001). ***I***, Maximum intensity projection of a 3D *z*-stack, illustrating an extended-duration movie highlighting Oregon Green 488 BAPTA-1-expressing myelinating oligodendrocytes (Movies 1 and 2). Scale bar, 100 μm. ***J***, Distribution of average frequency, duration, and amplitude of Ca^2+^ events per oligodendrocyte process. Comparison between AAV-mbp:jGCaMP8s and Oregon Green 488 BAPTA-1 AM indicator. A two-tailed statistical unpaired *t* test was used from 315 (AAV-mbp:jGCaMP8s) and 217 (Oregon Green) oligodendrocyte processes, analyzed from *N* = 10 (AAV-mbp:jGCaMP8s) and *N* = 15 (Oregon Green) movies. ****p ≤ 0.0001; ns, not significant.

It is well established that primary cultures of OPCs, in the absence of neurons, are capable of differentiating and synthesizing MBP, a protein produced solely by mature oligodendrocytes (Friess et al., 2016). Oligodendrocyte cultures were infected with AAV-mbp:jGCaMP8s and maintained in culture for 4 d prior to live Ca^2+^ imaging at DIV8. We detected eGFP expression in >70% of MBP^+^ oligodendrocytes (Fig. 2*A*; Extended Data Fig. 2-1). Our cultures, primarily consisting of enriched OPCs populations, were derived from mixed glial cultures; therefore, to assess the specificity of our construct, we excluded the expression of AAV-mbp:jGCaMP8s in glial fibrillary acidic protein (GFAP)^+^ astrocytes. As illustrated in Figure 2*B*, eGFP^+^ cells exhibit no colocalization with GFAP^+^ cells.

10.1523/ENEURO.0540-23.2024.f2-1 Figure 2-1 **A.** Representative image of myelinating oligodendrocytes (DIV8) showing eGFP expression for jGCaMP8  s (green) and mature oligodendrocytes (MBP, red). Nuclei (white) were labeled with DAPI dye. Scale bar: 100 μm. **B.** Quantification of eGFP and MBP cells per µm^2^ (left panel) and percent of MBP^+^/eGFP^+^ cells (right panel). This analysis was performed using maximum intensity projections of 3D z-stack images and quantification was obtained by using the “Analyze particles'‘ function of FIJI ImageJ software; N = 3 oligodendrocytes cultures. Download Figure 2-1, TIF file.

By live imaging, we recorded spontaneous Ca^2+^ episodes localized in myelinating processes (Fig. 2*C*, arrows, *D*; Movies 1 and 2), consistently with prior reports (Rui et al., 2020). [Ca^2+^]_i_ events exhibited substantial heterogeneity in frequency, duration, and amplitude values among oligodendrocyte processes (Fig. 2*E*–*G*). A positive correlation between duration and amplitude of Ca^2+^ events was observed, namely, longer oscillations displayed at higher amplitudes (Fig. 2*H*).

We compared the efficiency of AAV-mbp:jGCaMP8s and Oregon Green 488 BAPTA-1 AM, a commonly used Ca^2+^ imaging dye, to measure [Ca^2+^]_i_ signaling. In oligodendrocytes cultures, Ca^2+^ events detected by AAV-mbp:jGCaMP8s displayed comparable dynamic features as those detected by Oregon Green, using the same acquisition time frame and exposure time (>1 s), but the rAAV strategy improved the oligodendrocyte processes spatial resolution (compare Fig. 2*C*,*I*,*J*; Movies 1 and 2). Overall, these results demonstrate the efficacy and specificity of the developed rAAV, allowing detection of Ca^2+^ signals even from microdomains within the cell processes, thereby providing an experimental improvement in monitoring Ca^2+^ dynamics in myelinating oligodendrocytes.

### Ca^2+^ dynamics in oligodendrocytes during spinal slice culture development

**Figure 3.**
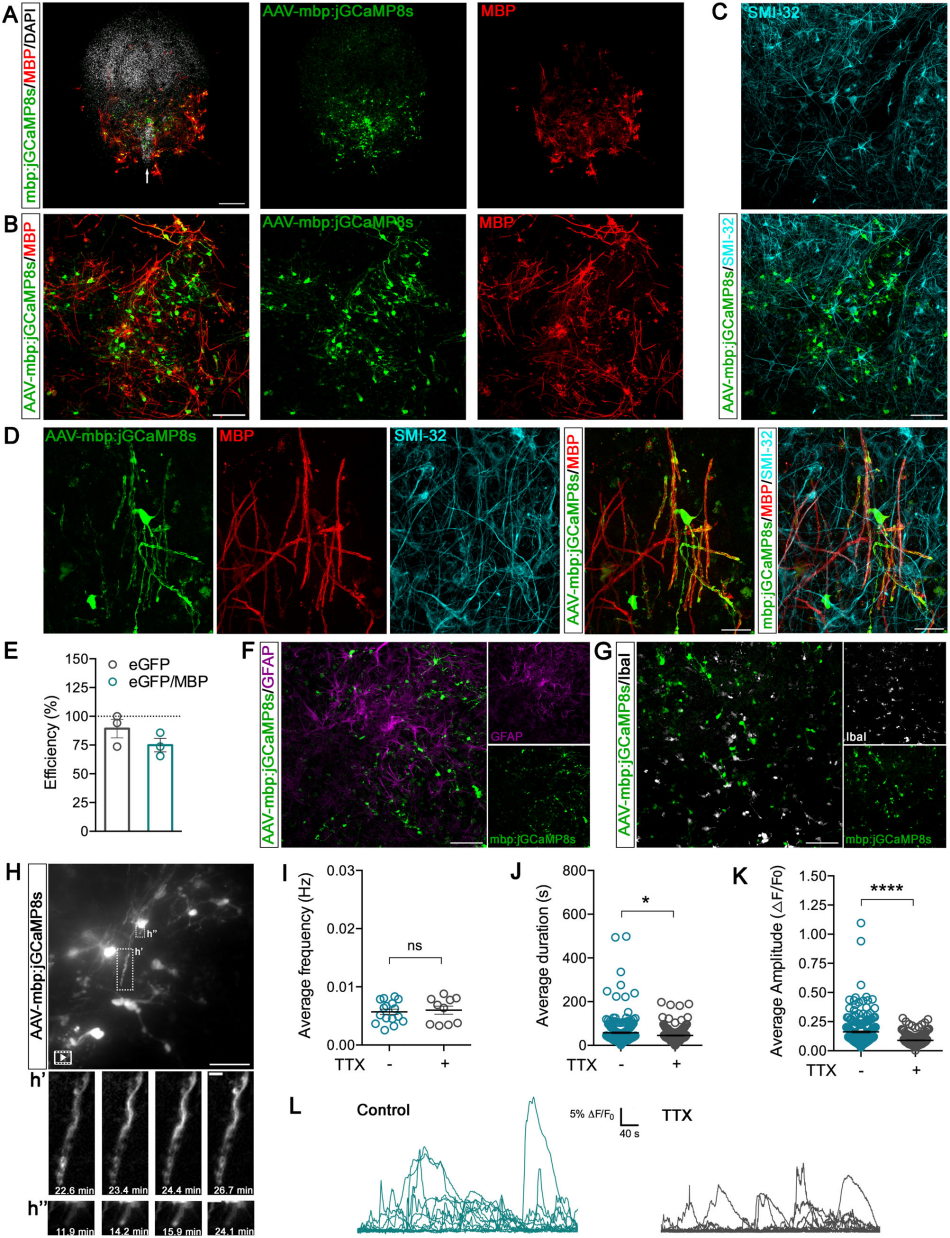
Ex vivo AAV-mbp:jGCaMP8s screening in spinal cord organ cultures at DIV14. ***A***, Representative image of the whole spinal organotypic culture showing AAV-mbp:jGCaMP8s expression (green) and mature oligodendrocytes (MBP, red). Nuclei (white) were labeled with DAPI dye. Note the ventral region highlighted by the ventral fissure (arrow). Scale bar, 300 μm. ***B***, Representative higher magnification image of the spinal ventral region (same as in ***A***) showing AAV-mbp:jGCaMP8s expression (green) and mature oligodendrocytes (MBP, red). Scale bar, 100 μm. See Extended Data Figure 3-1 for more details. ***C***, Representative confocal micrograph of spinal organotypic ventral region showing neurons (SMI-32, cyan) and AAV-mbp:jGCaMP8s expression (green). Scale bar, 100 μm. ***D***, Representative higher magnification images of spinal cord organotypic cultures showing AAV-mbp:jGCaMP8s expression (green), mature oligodendrocytes (MBP, red), and neurons (SMI-32, cyan). Scale bar, 30 μm. ***E***, Quantification of the AAV-mbp:jGCaMP8s efficiency in spinal cord organotypic cultures at DIV14. Data are represented as percentages. The analysis was performed using maximum intensity projections of 3D *z*-stack images, and quantification was obtained by using the “Analyze particles” function of the Fiji ImageJ software; *N* = 3 spinal cord organotypic cultures. ***F***, Representative image of ventral region of spinal cord organotypic cultures showing AAV-mbp:jGCaMP8s expression (green) and GFAP^+^ astrocytes (magenta). Scale bar, 100 μm. ***G***, Representative image of ventral region (same as in ***F***) showing AAV-mbp:jGCaMP8s expression (green) and microglia (IbaI, white). Scale bar: 100 μm. ***H***, Maximum intensity projection of a 3D *z*-stack, illustrating an extended-duration movie highlighting jGCaMP8s-expressing myelinating oligodendrocytes. Scale bar, 100 μm. Frames from the time-lapse imaging are indicated with a white box corresponding to panel ***h*’**, ***h*’’**; scale bar, 10 μm. ***I***, Average distribution of Ca^2+^ wave frequencies (Hz) per oligodendrocyte process before and after application of TTX. ***J***, Average distribution of Ca^2+^ wave duration per oligodendrocyte process before and after application of TTX. ***K***, Average distribution of Ca^2+^ amplitude per oligodendrocyte process before and after application of TTX. ***L***, Representative traces of Δ*F* / *F*_0_ over time before and after application of TTX. Scale bar, 40 s, and 5% Δ*F* / *F*_0_. Two hundred twenty (control) and one hundred thirty-five (TTX) oligodendrocyte processes, analyzed from *N* = 16 (control) and *N* = 10 (TTX) movies. Two-tailed statistical tests used: unpaired parametric *t* test in ***I*** and Mann–Whitney in ***J*** and ***K***. *p < 0.05; ***p ≤ 0.001; ns, not significant. See Extended Data Figures 3-2–3-4 and Movies 3 and 4 for more details.

We exploited AAV-mbp:jGCaMP8s specificity in organotypic spinal cord cultures, where sensory, motor, and interneuron circuit development are accompanied by resident glia cell progressive maturation (Avossa et al., 2003). Spinal slices were administered with AAV-mbp:jGCaMP8s at DIV7 and imaged at DIV14. Immunostaining with MBP antibody confirmed that the rAAV expression was specific to myelinating oligodendrocytes; accordingly, eGFP^+^ cells exhibited the unique oligodendrocyte localization and morphology (Fig. 3*A*,*B*), while neurons, marked by SMI-32, a neurofilament marker, were not eGFP^+^ (Fig. 3*C*). This becomes notably clearer when examining confocal images at higher magnification of SMI-32^+^ axons ensheathed by eGFP^+^ and MBP^+^ processes (Fig. 3*D*; Extended Data Fig. 3-1). Quantification of MBP^+^/eGFP^+^ cells further supported the specificity of rAAV expression in myelinating oligodendrocytes (Fig. 3*E*), without significantly affecting cell death (Extended Data Fig. 3-2). Staining with GFAP and Iba1, targeting astrocytes and microglia, respectively, confirmed the absence of colocalization with eGFP^+^ cells (Fig. 3*F*,*G*). Collectively, this data indicates that AAV-mbp:jGCaMP8s in spinal cultures selectively targets myelinating oligodendrocytes while excluding neurons and other resident glial cell types.

10.1523/ENEURO.0540-23.2024.f3-1 Figure 3-1 **A.** Representative image of spinal cord organotypic cultures showing AAV-mbp:jGCaMP8  s expression (green), mature oligodendrocytes (MBP, red) and neurons (SMI-32, cyan). Scale bar: 30 μm. Inset represents an image at higher magnification of the region marked in the white box. **B.** Histogram of MBP, SMI-22 and eGFP (AAV-mbp:jGCaMP8  s) intensity levels along the dashed line indicated in A. Download Figure 3-1, TIF file.

10.1523/ENEURO.0540-23.2024.f3-2 Figure 3-2 **A.** Representative image of spinal cord organotypic cultures at DIV14 comparing control (left panel) and AAV-mbp:jGCaMP8s-infected samples (right panel, green) with propidium iodide (PI, red). Nuclei (white) were labeled with DAPI dye. Scale bar: 100 μm. **B.** Quantification of PI and PI/eGFP cell numbers relative to area. **C.** Representative image of spinal cord organotypic cultures at DIV21 comparing control (left panel) and AAV-mbp:jGCaMP8s-infected samples (right panel, green) with PI (red). Nuclei (white) were labeled with DAPI dye. Scale bar: 100 μm. **D.** Quantification of PI and PI/eGFP cell numbers relative to area. The number of PI or PI/eGFP-labeled cells was determined using the “Analyze particles'‘ function of FIJI ImageJ software. Control, N = 3; AAV-mbp:jGCaMP8  s, N = 3 spinal cord organotypic cultures. Download Figure 3-2, TIF file.

10.1523/ENEURO.0540-23.2024.f3-3 Figure 3-3 **A.** Maximum intensity projection illustrates a simultaneous neuron whole-cell recording and jGCaMP8s-expressing myelinating oligodendrocytes (green). **B.** Representative traces of spontaneous neuronal activity recorded from control (green) and upon TTX treatment (gray); insets show abolishment of both individual and clustered spontaneous post-synaptic currents by TTX, with only miniature post-synaptic currents left. **C.** Representative traces of oligodendrocytes Ca^2+^ activity expressed as ΔF/F0 over time recorded simultaneously with neuronal activity. **D.** Distribution of average Ca^2+^ wave frequency, duration and amplitude of 31 [Control] and 27 [TTX] oligodendrocyte processes, analyzed from N = 4 [Control] and N = 3 [TTX] movies. Two-tailed statistical Mann–Whitney test was used; *P < 0.05; ns, not significant. Download Figure 3-3, TIF file.

**Figure 4.**
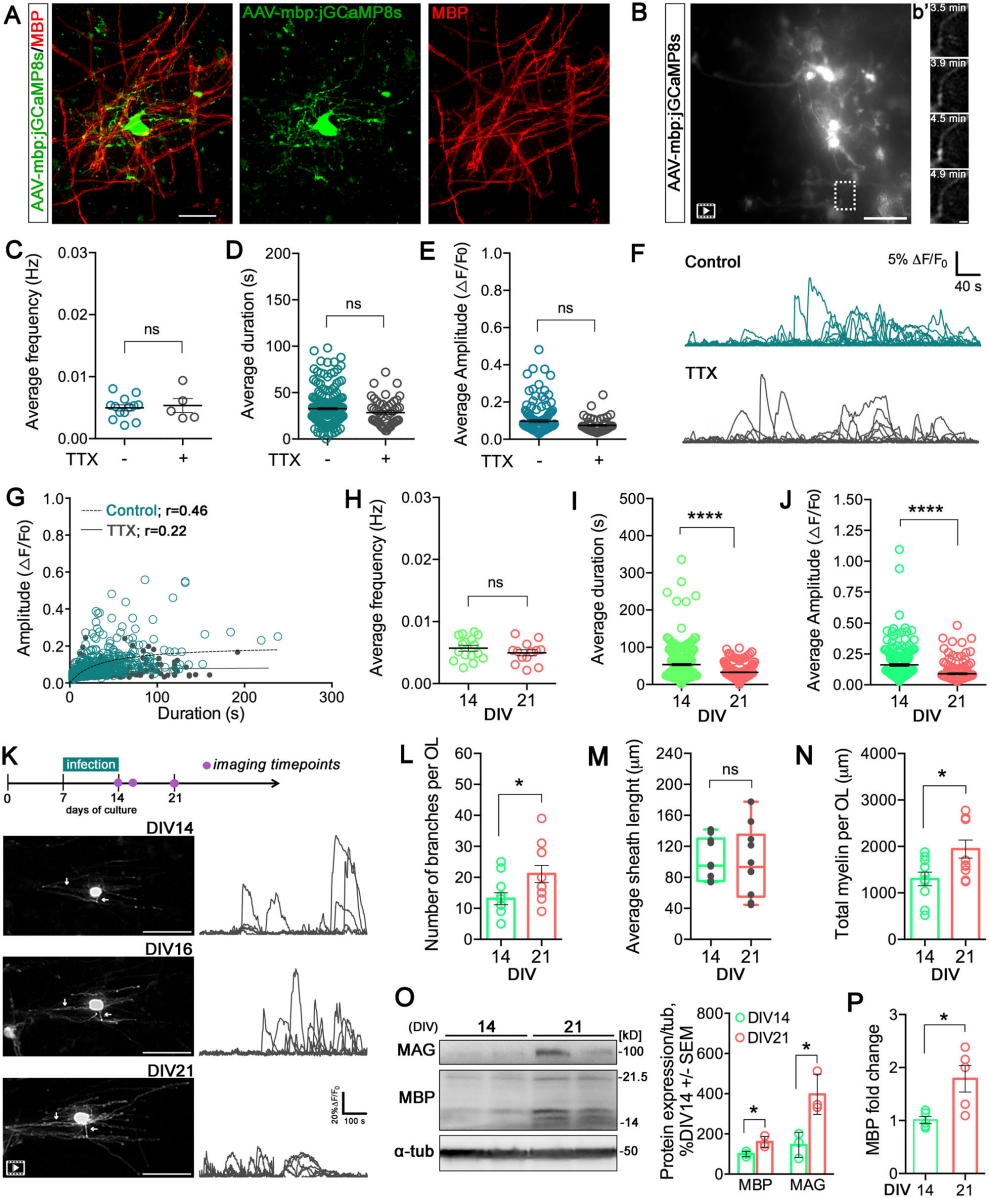
Ex vivo AAV-mbp:jGCaMP8s screening in spinal cord organ cultures at DIV21. ***A***, Representative image of the ventral region of spinal cord showing AAV-mbp:jGCaMP8s expression (green) and mature oligodendrocytes (MBP, red). Scale bar, 30 μm. See Extended Data Figure 4-1 for more details. ***B***, Maximum intensity projection of a 3D *z*-stack, illustrating an extended-duration movie highlighting jGCaMP8s-expressing myelinating oligodendrocytes. Scale bar, 100 μm. Frames from the time-lapse imaging are indicated with a white box corresponding to panel ***b*’**; scale bar, 10 μm. ***C***, Distribution of average Ca^2+^ wave frequencies (Hz) of 183 (control) and 49 (TTX) oligodendrocyte processes, analyzed from *N* = 13 (control) and *N* = 5 (TTX) movies. A two-sided statistical unpaired parametric *t* test was used, ns; not significant. ***D***, Distribution of average Ca^2+^ wave duration of 183 (control) and 49 (TTX) oligodendrocyte processes, analyzed from *N* = 13 (control) and *N* = 5 (TTX) movies. A two-tailed statistical Mann–Whitney test was used; ns, not significant. ***E***, Distribution of average Ca^2+^ amplitude of 183 (control) and 49 (TTX) oligodendrocyte processes, analyzed from *N* = 13 (control) and *N* = 5 (TTX) movies. A two-tailed statistical Mann–Whitney test was used; ns, not significant. ***F***, Representative traces of Δ*F* / *F*_0_ over time before and after application of TTX. Scale bar, 40 s, and 5% Δ*F* / *F*_0_. ***G***, Correlation between amplitude and duration of individual Ca^2+^ events of 183 (control) and 49 (TTX) oligodendrocyte processes, analyzed from *N* = 13 (control) and *N* = 5 (TTX) movies [Pearson's correlation, *r* = 0.46; *p* < 0.0001 (control); *r* = 0.22; *p* = 0.005 (TTX); Movies 5, 6]. ***H***, Distribution of average Ca^2+^ events frequencies (Hz) between 220 (DIV14) and 183 (DIV21) oligodendrocyte processes, analyzed from *N* = 16 (DIV14) and *N* = 13 (DIV21) movies. A two-tailed statistical unpaired parametric *t* test was used; ns, not significant. ***I***, Distribution of average Ca^2+^ events duration between 220 (DIV14) and 183 (DIV21) oligodendrocyte processes, analyzed from *N* = 16 (DIV14) and *N* = 13 (DIV21) movies. A two-tailed statistical Mann–Whitney test was used; ****p < 0.0001. ***J***, Distribution of average Ca^2+^ events amplitude between 220 (DIV14) and 183 (DIV21) oligodendrocyte processes, analyzed from *N* = 16 (DIV14) and *N* = 13 (DIV21) movies. A two-tailed statistical Mann–Whitney test was used; ****p < 0.0001. ***K***, Experimental timeline of Ca^2+^ imaging for the ex vivo longitudinal study tracking myelinating oligodendrocytes at DIV14, DIV16, and DIV21. White arrows indicate sheaths that became longer, over 7 d. In the right panel, oligodendrocyte Ca^2+^ traces for three developmental stages showing traces recorded sequentially in different single-cell processes (Movies 7–9). Scale bar, 100 s, and 20% Δ*F* / *F*_0_. Experiments were independently repeated three times. See Extended Data Figure 4-2 for more details. ***L***, Quantification of the number of branches at DIV14 and DIV21. Points represent individual oligodendrocytes. A two-tailed statistical unpaired *t* test was used; *p ≤ 0.05. ***M***, Quantification of myelin sheaths length at DIV14 and DIV21. Points represent the average of myelin sheaths of individual oligodendrocytes. A two-tailed statistical unpaired *t* test was used; ns, not significant. ***N***, Quantification of total myelin [(mean sheath length) × (number of sheaths per oligodendrocyte)] at DIV14 and DIV21. The number of branches, myelin sheath length, and total myelin sheath per oligodendrocyte were determined using the “Neuroanatomy SNT neuronal tracer” plugin of the Fiji ImageJ software, analyzed from *N* = 11 (DIV14) and *N* = 12 (DIV21) oligodendrocytes from seven spinal cord organotypic cultures. A two-tailed statistical unpaired *t* test was used; *p ≤ 0.05. ***O***, Immunoblot analysis of MBP and MAG protein levels. Quantification of MBP and MAG levels at DIV21 relative to DIV14 is shown, analyzed from *N* = 3 (DIV14) and *N* = 3 (DIV21) spinal cord organotypic cultures. A two-tailed statistical unpaired parametric *t* test was used; *p ≤ 0.05. ***P***, mRNA levels of MBP validated by qPCR, analyzed from *N* = 5 (DIV14) and *N* = 5 (DIV21) spinal cord organotypic cultures. A two-tailed statistical Mann–Whitney test was used; *p < 0.05.

10.1523/ENEURO.0540-23.2024.f3-4 Figure 3-4 Correlation between amplitude and duration of individual Ca^2+^ events in organotypic spinal cord cultures at DIV14 from 220 [Control] and 135 [TTX] oligodendrocyte processes, analyzed from N = 16 [Control] and N = 10 [TTX] movies. Pearson’s correlation coefficient was calculated using the “Coloc 2'‘ plug-in of FIJI ImageJ software. Two-tailed statistical unpaired *t*-test was used; r = 0.05, P < 0.13 [Control]; r = 0.03, P < 0.36) [TTX]. Download Figure 3-4, TIF file.

Based on these findings, we performed live Ca^2+^ imaging in spinal cord tissues at DIV14, detecting Ca^2+^ events localized in oligodendrocytes processes (Fig. 3*H*,*L*) and measuring the average values of frequency, duration, and amplitude (Fig. 3*I*–*K*; Movies 3 and 4). Since neuronal activity might modulate [Ca^2+^]_i_ signaling in oligodendrocytes (Pitman and Young, 2016), we selectively blocked fast voltage-gated Na^+^ channels by applying TTX (1 μM) to the spinal cultures (Panattoni et al., 2021). TTX treatment roughly halved the Ca^2+^ events amplitude in oligodendrocytes processes while significantly decreasing their duration without affecting the average Ca^2+^ frequency (Fig. 3*I*–*L*; Movies 3 and 4). Simultaneous recording of oligodendrocytes Ca^2+^ signaling and electrophysiology in neurons allowed us to further confirm these findings (Extended Data Fig. 3-3). In both the control and TTX-treated slices, we did not observe a significant correlation between Ca^2+^ amplitude and duration (Extended Data Fig. 3-4).

During differentiation, oligodendrocytes might undergo profound changes in cellular interactions, neurotransmitter receptor expression, and their overall response to neuronal activity (Clemente et al., 2013; Moura et al., 2022). We tracked Ca^2+^ activity at a later stage of development, namely, at DIV21 to explore whether distinct Ca^2+^ signals are generated by oligodendrocytes during spinal circuit maturation (Fig. 4*A*; Rosato-Siri et al., 2004). Immunohistochemical analysis showed that >80% of the eGFP+ cells colabeled with MBP, while no colocalization was detected with neurons and other glial cells (Extended Data Fig. 4-1). We investigated the effect of neuronal activity on oligodendrocyte Ca^2+^ dynamics in spinal cord cultures at DIV21 (Fig. 4*B*–*F*). Our analysis showed no significant alterations in the average frequency, duration, and amplitude of Ca^2+^ events when neuronal action potentials were inhibited by TTX (Fig. 4*C*–*F*; Movies 5 and 6). Differently from DIV14, we observed a significant positive correlation between amplitude and duration of Ca^2+^ signals in both the control and TTX conditions (Fig. 4*G*).

10.1523/ENEURO.0540-23.2024.f4-1 Figure 4-1 **A.** Representative image of the whole spinal organotypic culture at DIV21 showing AAV-mbp:jGCaMP8  s expression (green) and mature oligodendrocytes (MBP, red). Nuclei (white) were labeled with DAPI dye. Note the ventral region highlighted by the ventral fissure (arrow). Scale bar: 300 μm. **B.** Representative higher magnification image of the spinal ventral region (same as in **A**) showing AAV-mbp:jGCaMP8  s expression (green), mature oligodendrocytes (MBP, red) and neurons (SMI-32, cyan). Scale bar: 30 μm. **C.** Quantification of the AAV-mbp:jGCaMP8  s efficiency in spinal cord organotypic cultures at DIV21. Data are represented as percentages. The analysis was performed using maximum intensity projections of 3D z-stack images, and quantification was obtained by using the “Analyze particles'‘ function of FIJI ImageJ software; N = 3 spinal cord organotypic cultures. **D.** Representative image of the ventral region of spinal cord showing AAV-mbp:jGCaMP8  s expression (green) and GFAP^+^ astrocytes (magenta). Scale bar: 100 μm. **E.** Representative higher magnification image of spinal cord showing AAV-mbp:jGCaMP8  s expression (green), mature oligodendrocytes (MBP, red) and GFAP^+^ astrocytes (magenta). Scale bar: 30 μm. **F.** Representative image of ventral region of spinal cord showing AAV-mbp:jGCaMP8  s expression (green) and microglia (IbaI, white). Scale bar: 100 μm. Download Figure 4-1, TIF file.

10.1523/ENEURO.0540-23.2024.f4-2 Figure 4-2 Representative image of spinal cord organotypic cultures showing AAV-mbp:jGCaMP8  s expression (green) and PI (red). The arrow indicates the cell that was recorded in the longitudinal *in vivo* study represented in Figure 4  K. Scale bar: 100 μm. Download Figure 4-2, TIF file.

**Movie 1:** Myelin Ca^2+^ events in primary oligodendrocyte cultures infected with AAV-mbp:jGCaMP8s **(1)** [view online] and administered with Oregon Green 488 BAPTA-1 A.M. **(2)** [view online], related to Figure 2*C* and Figure 2*I*, respectively.

**Movie 2.** Myelin Ca^2+^ events in primary oligodendrocyte cultures infected with AAV-mbp:jGCaMP8s **(1)** [view online] and administered with Oregon Green 488 BAPTA-1 A.M. **(2)** [view online], related to Figure 2*C* and Figure 2*I*, respectively.

**Movie 3.** Myelin Ca^2+^ events in an organotypic spinal cord culture at DIV14 infected with AAV-mbp:jGCaMP8s **(3)** [view online] and treated with TTX **(4)** [view online], related to Figure 3*H*.

**Movie 4.** Myelin Ca^2+^ events in an organotypic spinal cord culture at DIV14 infected with AAV-mbp:jGCaMP8s **(3)** [view online] and treated with TTX **(4)** [view online], related to Figure 3*H*.

**Movie 5.** Myelin Ca^2+^ events in an organotypic spinal cord culture at DIV21 infected with AAV-mbp:jGCaMP8s **(5)** [view online] and treated with TTX **(6)** [view online], related to Figure 4*B*.

**Movie 6.** Myelin Ca^2+^ events in an organotypic spinal cord culture at DIV21 infected with AAV-mbp:jGCaMP8s **(5)** [view online] and treated with TTX **(6)** [view online], related to Figure 4*B*.

**Movie 7:** Ca^2+^ imaging for the ex vivo longitudinal study tracking myelinating oligodendrocytes at DIV14 **(7)** [view online], DIV16 **(8)** [view online], and DIV21 **(9)** [view online], related to Figure 4*K*.

**Movie 8.** Ca^2+^ imaging for the ex vivo longitudinal study tracking myelinating oligodendrocytes at DIV14 **(7)** [view online], DIV16 **(8)** [view online], and DIV21 **(9)** [view online], related to Figure 4*K*.

**Movie 9.** Ca^2+^ imaging for the ex vivo longitudinal study tracking myelinating oligodendrocytes at DIV14 **(7)** [view online], DIV16 **(8)** [view online], and DIV21 **(9)** [view online], related to Figure 4*K*.

Besides the changes in the amplitude/duration correlation and in TTX sensitivity over the period of oligodendrocyte in vitro maturation and myelin stabilization around axons, we directly compared the average values of frequency, amplitude, and duration of Ca^2+^ signals in the spinal cultures between DIV14 and DIV21. We observed a remarkable decline in both amplitude and duration of Ca^2+^ events as development progressed (Fig. 4*I*,*J*), while the frequency remained unaffected (Fig. 4*H*). Longitudinal live imaging highlighted dynamic changes in Ca^2+^ duration and amplitude of oligodendrocytes throughout development without affecting long-term viability (Fig. 4*K*; Extended Data Fig. 4-2), along with increased oligodendrocyte branching complexity at DIV21 (Fig. 4*L*; Movies 7–9). Although sheath length remained consistent (Fig. 4*M*), the greater sheath number at DIV21 resulted in a higher total amount of myelin per oligodendrocyte (Fig. 4*N*). Accordingly, expression levels of MBP and MAG proteins were higher at DIV21 compared with those at DIV14 (Fig. 4*O*), accompanied by elevated *Mbp* RNA expression (Fig. 4*P*). These findings suggest an enhanced myelinating capacity per oligodendrocyte during the development of organotypic spinal cord cultures. Overall, our results suggest a developmental regulation of [Ca^2+^]_i_ dynamics in oligodendrocytes with a transient dependence upon action potential firing.

## Discussion

Our major result is the successful characterization of a recombinant viral vector expressing the jGCaMP8s under the control of the MBP promoter. We exploited AAV-mbp:jGCaMP8s to visualize and monitor Ca^2+^ activity in myelinating oligodendrocytes in vitro and in ex vivo slice cultures. This model sheds light on the expression of MBP, a critical myelin protein (Chernoff, 1981), in concert with fluctuations in [Ca^2+^]_i_ during myelination. jGCaMP8s expression was histologically and functionally shown to be restricted to mature oligodendrocytes in both models, such a selectivity was particularly evident in spinal cord organotypic cultures, where numerous resident glial cells are present and eGFP^+^ oligodendrocytes tightly ensheath SMI32^+^ axons.

In recent studies, myelin Ca^2+^ activity was investigated in transgenic zebrafish and rodent animal models (Krasnow et al., 2018; Battefeld et al., 2019). While rAAVs are an easier and cost-effective approach, their utility is limited by the lack of natural AAV capsids with oligodendrocyte affinity. Our study successfully exploited the viral capsid Olig001 which displayed exceptional in vivo tropism for oligodendrocytes (Powell et al., 2016). The specificity of AAV-mbp:jGCaMP8s in myelinating oligodendrocytes was further reinforced by the absence of colocalization with microglia and neurons in organotypic cultures, as well as with astrocytes in both cultured models.

Comparing its kinetics to Oregon Green BAPTA-1, the current AAV-mbp:jGCaMP8s displayed a similar response, due to our imaging settings (Sun et al., 2013). However, the chemical probe showed a lower spatial resolution required for optimal measuring of Ca^2+^ signals in oligodendrocytes processes. The robust expression of AAV-mbp:jGCaMP8s in mature oligodendrocytes not only enabled precise imaging of Ca^2+^ events in myelin processes but also demonstrated its suitability for chronic and high-resolution Ca^2+^ signaling recordings without affecting total cell death. One methodological consideration is the sampling rate (∼0.03 Hz) employed for detecting Ca^2+^ events, determined by our imaging setup. This design limited our observations to Ca^2+^ events longer than the subsecond duration Ca^2+^ transients.

We focused on the development of our versatile tool to record the spatiotemporal pattern of Ca^2+^ changes in myelinating spinal organ cultures. We observed a substantial number of traced axons ensheathed by MBP protein, together with a highly active period of spontaneous Ca^2+^ events during the early postnatal week. At this age, individual oligodendrocytes make short myelin sheaths on multiple axons, retracting from some axons while maintaining sheaths on others (Czopka et al., 2013); this dynamic process is directly correlated with Ca^2+^ signals as demonstrated by Baraban et al. (2018). Up to now, Ca^2+^ changes in OPCs processes have largely been suggested as dependent on neuronal action potentials (Wake et al., 2011). Unexpectedly, our results indicated that the Ca^2+^ frequency was independent of action potential generation both at DIV14 and DIV21. Conversely, only at DIV14, Ca^2+^ amplitude and duration significantly decreased following the pharmacological blockade of neuronal activity, whereas no significant modulation was observed at DIV21. These data suggest that Ca^2+^ events in oligodendrocytes can be sustained by both neuronal activity and intrinsic mechanisms (Hamilton et al., 2017; Rui et al., 2020; Fiore et al., 2022). In particular, myelination regulates the influx of extracellular Ca^2+^ and its uptake from endoplasmic reticulum and mitochondria depending on the developmental stage (Krasnow et al., 2018; Paez and Lyons, 2020; Meyer and Rinholm, 2021). The morphology, density, and spatial distribution of mitochondria reflect the modulation of energy demand and Ca^2+^ buffering capacity in oligodendrocytes during development. Mature oligodendrocytes are sustained by glycolysis and characterized by smaller and fewer mitochondria. In contrast, OPCs and developing oligodendrocytes have a higher metabolic demand, exhibiting a dense population of elongated mitochondria. These cells use both glycolysis and oxidative phosphorylation to produce energy for lipid biosynthesis and active myelination (Kirischuk et al., 1995; Rinholm et al., 2016; Rone et al., 2016; Meyer and Rinholm, 2021; Narine and Colognato, 2022). Our results showed a stage-dependent decline in the Ca^2+^ amplitude and duration during myelination. Specifically, spinal slices at DIV14 display heightened amplitude and prolonged duration compared with those at DIV21, without alterations in the frequency of Ca^2+^ events. Interestingly, large-amplitude of Ca^2+^ waves occurred primarily between DIV13 and DIV15 in brain slices, coinciding with a phase of rapid longitudinal remodeling of myelin (Battefeld et al., 2019).

Our results suggest a decreased energy demand from oligodendrocytes with age, indicating possible modifications in mitochondrial morphology, density, and Ca^2+^ buffering capacity, all issues needing further investigations. Interestingly, the absence of correlation between Ca^2+^ events amplitude and duration at DIV14 in spinal organ slices, observed in both the control and TTX conditions, could be attributed to the fact that among MBP-expressing cells some are wrapping axons, while others are maintaining myelin. These simultaneous activities reveal a heterogeneous energy demand and Ca^2+^ buffering capacity among oligodendrocytes, accompanied by a combined glycolysis-OXPHOS ATP production. In this phase, neuronal activity partially contributes to Ca^2+^ buffering capacity, underscoring that myelination may also be regulated by additional Ca^2+^-mediated pathways. However, at DIV21, the correlation between Ca^2+^ amplitude and duration was recovered, potentially due to the majority of axons being already myelinated and oligodendrocytes being mainly involved in maintaining myelin sheaths with a low ATP production through the glycolytic pathway. This reliance on glycolysis could result in a similar Ca^2+^ buffering capacity among mitochondria. Thus, Ca^2+^ dynamics in organ cultures between DIV14 and DIV21 align with significant morphological changes in oligodendrocytes, evidenced by enhanced myelin production per oligodendrocyte, increased number of branches, elevated expression of MBP and MAG proteins, and augmented levels of MBP mRNA expression levels as development progress. Taken together, our study provides new evidence regarding the role of Ca^2+^ signaling in mediating different stages of myelination.

Ca^2+^ cellular sources and signaling pathways in mature oligodendrocytes remain poorly understood (Thornton and Hughes, 2020) and need further explorations. Nonetheless, the employment of AAV-mbp:jGCaMP8s with high-resolution Ca^2+^ imaging, coupled with the manipulation of neuronal activity, not only enhances the spatiotemporal resolution of the recordings but also holds the potential to accelerate our understanding of how Ca^2+^ intrinsically regulates oligodendrocyte myelination.

## Synthesis

Reviewing Editor: Silvia Pagliardini, University of Alberta Decisions are customarily a result of the Reviewing Editor and the peer reviewers coming together and discussing their recommendations until a consensus is reached. When revisions are invited, a fact-based synthesis statement explaining their decision and outlining what is needed to prepare a revision will be listed below. The following reviewer(s) agreed to reveal their identity: Michal Hetman, Pascal Branchereau.

The authors have thoroughly addressed the concerns raised by the reviewers.

Minor concern on the so-called simultaneous live imaging of oligodendrocytes and single cell electrophysiology recordings from spinal neurons. If I understand correctly the newly added figure 3.3 does not show simultaneous recordings but recordings performed on the same preparation. This must be clearly stated in the revised manuscript. If simultaneous recordings were performed, then they must be shown in order to highlight if neurons get depolarized when adjacent oligodendrocytes exhibit Ca2+ events.

Figure 3.3 must be edited because the panel D is partly masking the current recording.

Remark: that was complicated for me to apprehend the figures labelling, some figures being labelled by letters and others by numbers. Please make figure labelling similar across figures.

## Author Response

We are grateful to the Editor and Reviewers for the time and effort dedicated to evaluating our manuscript and for the opportunity to re-submit a fully revised version of our manuscript (MS) "Exploring Ca2+ dynamics in myelinating oligodendrocytes through rAAV-mediated jGCaMP8s expression in developing spinal cord organ cultures" (eN-MNT-0540-23), that we hope can be now suitable for publication in the eNeuro journal.

We have carefully revised our work addressing all the points raised by the Reviewers. This has implied adding new results obtained by 6 additional experimental series (now shown in the new panels of Figures 3I, Figure 4K-P and in Figures 3-2, 3-3, 4-1 and 4-2), including physiological data aiming at reinforcing our results. All new experiments and measures have been added to the revised MS as detailed in the point-by-point response below. Your valuable comments and suggestions have greatly contributed to enhancing the quality of the paper.

In our study we exploit the recombinant adeno-associated virus (rAAV)/Olig001 capsid to express the genetically encoded calcium (Ca2+) indicator jGCaMP8s under the control of the myelin basic protein (MBP) promoter, and we challenged the specificity and selectivity of the newly developed tool to explore the dynamics of Ca2+ signaling in two distinct rodent models: primary cultures of oligodendrocytes and organotypic spinal cord slices, where we could monitor and visualize Ca2+ events in oligodendrocyte processes with excellent spatiotemporal resolution. Our findings contribute to understanding the modulation of myelin Ca2+ signaling in oligodendrocytes throughout development and we believe hold the potential to offer invaluable insights for live monitoring of oligodendrocytes Ca2+ signaling in health and disease.

This MS has not been previously published and is not currently under consideration by any other journal. All authors have approved the revised MS and have agreed to the eNeuro's submission policies. The authors have no conflicts of interest, financial or otherwise to declare.

We have uploaded a marked-up version of the MS, blue colored font the specific text changes and additional references made in response to each comment.

All MS's changes are itemized in the hereunder point-by-point response keyed to the Editor/Referee's comments.

We have included physiological experiments using electrophysiology in the revised MS, which were carried out by Luca Recupero. All authors are in agreement to include him as a co-author of the publication.

Best regards, Point-by-point response This is an interesting manuscript that reports a new live imaging tool for Ca signaling analysis in oligodendrocytes applied to organotypic spinal cord cultures from rat embryos.

The present paper shows interesting results but physiological data must be reinforced in order to demonstrate that the frequency of Ca2+ events drops while myelinization occurs. Leveraging their organotypic slice preparation, such a demonstration should be possible if the authors use techniques such as electrophysiology.

Response to comment 1: We were pleased to hear that the Reviewers find our MS interesting. We have followed the Reviewers' suggestions and reinforced the MS with additional physiological data, whenever possible (see new panels in Figure 4K-N and Figure 3-3). We feel relevant to clarify that in our experiments we did not report a significant change in the frequency of Ca2+ events along with increased myelination (see example in Figure 4C and H), what we have observed is a significant decline in the duration and amplitude of Ca2+ signals, specifically between DIV14 and DIV21 development (see Figure 4I-J). Importantly, with an additional series of experiments, we show now that these changes are concomitant with an increase in the levels of MBP and MAG proteins, as reinforced also by our new measures detecting an increase in MBP mRNA from DIV14 to DIV21 organotypic spinal cord cultures (see new Figure 4O-P in the revised MS; page 11, lines 363-6). In order to address this point with a physiological approach, we have added to the revised MS a longitudinal in vivo imaging study where we tracked single oligodendrocyte Ca2+ activity at different time points (see new Figure 4K). Our new experimental series and analysis unveiled an enhancement in myelin production per oligodendrocyte and increased branching complexity when comparing DIV14 with DIV21 (see Figure 4L-N of the revised MS). Altogether, our findings and additional experiments strongly support a progressive increase in total myelination in our model during in vitro development and provide insights into oligodendrocytes physiological changes in Ca2+ signaling during myelination. We have now emphasized these results explicitly in the revised MS (results page 11, lines 359-66 and discussion page 13, lines 435-40).

Abstract:

- The authors speak about "myelin Ca2+ events" but do not bring elements toward a myelinating role for oligodendrocytes. Do the authors speak about myelination only because of the presence of myelin basic protein (MBP)? Response to comment 2: The term "myelin Ca2+ events", is used to describe the Ca2+ activity in myelinating oligodendrocytes, and their role in myelination is now more clearly stated in the revised abstract (page 1, lines 3-4). Briefly in our study, we used the genetically encoded calcium indicator jGCaMP8s under the control of MBP promoter to visualize Ca2+ activity in myelinating oligodendrocytes. Indeed, Ca2+ imaging was performed on the mice spinal cord explants at DIV14 and DIV21 culture period, namely during the maturation of myelin sheaths (Avossa et al., 2003; see Figure 6 and text herein). In our investigation we used MBP as a crucial marker for identifying oligodendrocyte differentiation and myelination in the CNS; quantifying MBP is a generally accepted index of myelination (Mathisen et al., 1993; Sternberger et al., 1978; Chernoff G F., 1981). Myelination by oligodendrocytes is now further supported by the new set of experiments quantifying MBP and MAG proteins, MBP mRNA levels, enhancement in myelin production per oligodendrocytes and, in a longitudinal study, we now report increased oligodendrocyte branching complexity along with changes in Ca2+ dynamics, when comparing DIV14 with DIV21 (see Figure 4L-P in the revised MS). Importantly, our study aligns with findings in Shiverer mice, where mutations in the Mbp gene result in significant hypomyelination in the CNS (Chernoff GF., 1981), underscoring the pivotal role of MBP in the myelination process as discussed in our investigation (page 11, lines 374-6).

- "..., the dynamics of Ca2+ events is governed by axon action potential firing". Firing coming from which cells? Nothing is said about the axon-myelin interface in the abstract.

Response to comment 3: We are grateful for this insightful inquiry regarding the dynamics of Ca2+ events and their association with axon action potential firing. In our study, we focused on the ventral spinal cord, and the action potential firing primarily stems from motoneurons, both directly and indirectly through premotor interneurons. However, we acknowledge the challenge posed by the use of TTX as a blocking agent, affecting all neurons, and preventing selective blocking of specific neuron types. Despite this limitation, our immunofluorescence experiments have revealed compelling evidence of close proximity of SMI32 positive axons and eGFP+ oligodendrocytes processes (Figure 3-1), indicating a notable association between SMI32 positive-axons and myelin within the ventral area of the cultured slices. SMI32 is an antibody against the dephosphorylated epitope on NF-H carboxyl terminal region and its selective capacity to mark motoneurons has been described in the spinal cord during development (Breckenridge et al., 1997; Tsang et al., 2000). This is in accordance with previous observations, where SMI32 cell staining has been frequently associated with myelination or size of emerging axons (Breckenridge et al., 1997; Kirkcaldie et al., 2002; see also Avossa et al 2003 for SMI32 and MBP in organotypic cultures). We have mentioned part of these insights into our revised MS to provide clarity on this particular point in the abstract (page 1, lines 15-17).

Results:

-A better definition of a "calcium event" is required. Some time lapse images illustrating such events could also be included in the main manuscript figures. Perhaps few examples of various "calcium events" could be presented in this manner.

Response to comment 4: We are grateful for this observation and for the suggestion to include time-lapse images in the main MS figures. Briefly, when we refer to "calcium events", we are primarily describing slow intracellular Ca2+ elevations events, with duration longer than 1 second. In the revised MS this parameter is more clearly reported in the methods section (page 7; lines 209-10). We have now included time-lapse images in the main MS figures to illustrate samples of Ca2+ events (Figure 2c', Figure 3h', h' and Figure 4b'). While we incorporated some representative time-lapse images, we have also included movie files as supplementary materials, which comprehensively demonstrate the dynamics of Ca2+ events. We believe this approach will favor the readers' understanding.

-As t-test has been used for data analysis, the authors should clarify whether normal distribution was tested for all analyzed parameters. Statistics are straightforward and correctly performed but many quantifications are lacking.

Response to comment 5: To ensure the validity of t-tests, data was tested for normality using the D'Agostino &Pearson omnibus normality test. Non-normally distributed data were tested by two-tailed Mann-Whitney test. We have updated the Materials and Methods section accordingly to explicitly mention the normality testing procedure (pages 8, 9; lines 276-80 of the revised MS). The results of these tests are summarized in Table 1, confirming that the data meet the assumptions of normal distribution necessary for the application of t-tests. Additionally, we have included in the revised MS all quantifications requested throughout the review process. Please note that each condition analyzed in every experiment account for samples coming from at least 3 different culture series as reported in the Methods section of the revised MS, unless otherwise stated (page 9; lines 282-3). Additionally, we reported the sample size information in Table 1 and in the figure legends.

- Please indicate how you did exclude the expression of AAV-mbp:jGCaMP8s in glial fibrillary acidic protein (GFAP)+ astrocytes.

Response to comment 6: To exclude the expression of AAV-mbp:jGCaMP8s in glial fibrillary acidic protein (GFAP)+ astrocytes, we conducted immunofluorescence analysis and measured the occurrence of co-localizations, as reported in the manuscript (page 9, lines 300-01; page 10, lines 328-9 and page 11, lines 347-8) in both cultured oligodendrocytes (Figure 2F) and organotypic spinal cultures (Figure 3F [DIV14], and Figure 4-1 [DIV21]).

- "..., namely longer oscillations displayed at higher amplitudes." What about the relationship between amplitude and frequency? Response to comment 7: We are grateful to the Reviewers for this useful comment. In our study, we meticulously examined calcium events associated with the myelin increase. We did not detect a positive correlation between frequency and amplitude of Ca2+ events both at DIV14 and DIV21 of development (in control slices as well as in TTX-treated ones) (see Figure I below). Nevertheless, the monitoring of these events is essential in explaining various stages of myelin development, maturation, stability, and degradation.

Figure I: Correlation between amplitude and frequency of individual Ca2+ events in organotypic spinal cord cultures at DIV14 and DIV21 in (left) control and (right) TTX-treated slices. DIV14: 220 [Control] and 135 [TTX] oligodendrocyte processes, analyzed from N= 16 [Control] and N= 10 [TTX] movies (Pearson r=0.096, P=0.054 [Control]; r=0.098, P=0.2669 [TTX]. DIV 21: 183 [Control] and 49 [TTX] oligodendrocyte processes, analyzed from N= 13 [Control] and N= 5 [TTX] movies (Pearson r=0.062, P=0.394 [Control]; r=0.7764, P=0.596 [TTX]).

- Figure 3: many AAVmbp:jGCaMP8s (eGFP+) cells are not MBP+. Can the authors explain why? This is not toward a rAAV expression specific to myelinating oligodendrocytes. A quantification of the amount of double labeled cells would be useful.

Response to comment 8: We thank the Reviewers for this comment and we agree that further clarification about this point is needed. The discrepancy noted in Figure 3, where many eGFP+ cells do not appear to be MBP+ cells, is due to the distinct subcellular expression patterns of the AAV-mbp:jGCaMP8s construct compared to endogenous MBP protein. Specifically, the AAV-mbp:jGCaMP8s exhibits higher expression in both processes and the soma, making nuclei more prominent. In contrast, MBP protein marked through immunofluorescence is predominantly expressed in processes while being lower in the soma. This discrepancy in expression patterns explain why some AAV-mbp:jGCaMP8s (eGFP+) cells may not appear to colocalize with MBP. Besides the different distribution of the two markers, the images collected were processed as z-stacks from 20× onwards, presenting only the maximum intensity projection approach. This method produces a single image with all the layers merged, showcasing maximum intensity, that may unveil processes that were not initially visible while still capturing soma expression. To address these considerations, we provided a quantification of eGFP+/MBP/DAPI cells at both DIV14 and DIV21 showing an efficiency of 75{plus minus}10% and 86{plus minus}19%, respectively (Figure 3E and Figure 4-1), confirming the specific expression of rAAV to myelinating oligodendrocytes.

We added a paragraph in the Methods section (pages 5, 6, lines 174-9) explaining the procedure used to assess this question, willing to enhance the clarity of our results.

- Unlike dissociated cultures, we did not observe statistically significant correlation between Ca2+ amplitude and duration (Suppl. Figure S2). Do the authors mean in the presence of TTX? Response to comment 9: Our data indeed indicates that there is no such correlation in both control and TTX (Figure 3-4). We have included this clarification in the revised text to ensure the accuracy of our statement (page 10, lines 340-1).

- Slice culture: the authors claim that all eGFP+ cells co-label with MBP but without any quantification it's difficult to make sure.

Response to comment 10: We agree with the Reviewers and we apologize for not having provided a clear quantification. We have now added in the revised MS a quantification of eGFP+ cells co-labeled with MBP. The results obtained, along with their statistical analysis, have been incorporated into Figure 3E (DIV14) and Figure 4-1 (DIV21).

- What is the use of assessing the correlation between amplitude and duration of Ca2+ signals? Response to comment 11: We are grateful to the Reviewers for giving us the opportunity to better address this issue. Examining the correlation between the amplitude and duration of Ca2+ signals, enables us to predict changes in myelination states, such as myelin retraction, remodeling, and growth (Baraban et al., 2018; Czopka et al. 2013; Krasnow et al., 2018). In our study, during initial myelination of SMI32+ axons (DIV14), we observed the absence of such a correlation in the early phases, together with the influence of action potential firing on the amplitude and duration of Ca2+ events. However, both features are lost at later stages (DIV21), suggesting potential variations in phases of myelin remodeling. This hypothesis is expanded in the discussion section (page 13, lines 417-22 and lines 431-5).

- The decline of Ca2+ events as development progressed is not correlated with any parameter indicating that myelinization is in progress (i.e., action potential propagation speed). Current results do not support this conclusion.

Response to comment 12: We appreciate these insightful comments and suggestions by the Reviewers. We detected a decline in the duration and amplitude of oligodendrocytes Ca2+ signals, specifically between DIV14 and DIV21 development, with no changes in the frequency of Ca2+events.

To compare myelination progress at these developmental time-points (DIV14 and DIV21) we have used MBP as a proxy of myelination given its pivotal role as the major structural element of CNS myelin, essential for both myelin wrapping and compaction (Readhead et al, 1987). We extended our study to quantify compact myelin using WB analysis and qPCR. Notably, MBP protein levels increased from DIV14 to DIV21, suggesting myelin initial wrapping and compaction (Figure 4O of the revised MS). This result is strengthened by the observed increase in the expression of MBP mRNA as the development of the cultures progressed (Figure 4P of the revised MS). It is important to highlight that MBP mRNA is transported to the growing tip of oligodendroglia processes (Ainger et al., 1993), with MBP translation induced at the innermost myelin layers, in contact with the axon.

In our longitudinal study, we now detect increased oligodendrocyte branching complexity and we report sequential imaging of Ca2+ activity in multiple myelin sheaths (from a number of different oligodendrocytes), which show substantial variations in Ca2+ amplitude and duration during development. In actively growing myelin sheaths (DIV14), both the amplitude and duration of Ca2+ events were substantially greater when compared to those at DIV21 (Figure 4 H-K). These observations strongly suggest a direct link between Ca2+ dynamics and the myelination process. Our conclusion is reinforced by the identification of an increase in total myelin sheaths and number of branches per oligodendrocyte at DIV21 (Figure 4L-M of the revised MS). We believe that incorporating this additional evidence will provide a more comprehensive understanding of the Ca2+ dynamics during the developmental myelination. This issue is now added in the results and discussion (page 11, lines 359-66 and page 13, lines 435-40, respectively).

The direct measure of the speed of action potential propagation in organotypic motoneuronal axons (during MBP development in vitro) could in principle be performed adopting the most recent strategies adapted for slice preparations, for example by combining single cell patch clamp (somatic) recordings and loose patch (axonal) recordings at different axonal locations, under direct optical control using a pipette-injected fluorophore (Kotler O. et al., 2023). Such an approach, is experimentally prevented in organotypic slices due to several factors: first, the deep location within the slice cell layers of motoneurons clusters, making them difficult target for successful patch clamp seal and recordings; second, the frequent entangled morphology of outgrowing motor axons in the ventral cord area (see Figure 3D of the current MS for SMI32 axons, for example), both conditions strongly limiting the anatomical access and positioning of recording pipettes at various distances from the cell soma in myelinated axons. In addition, to correctly visualize the in-vivo labeled motoneuron and its myelinated axon, a 2P microscopy is usually needed (Kotler O. et al., 2023). Besides, it would be virtually impossible to rule out the contribution of the increased excitable channels expression during neuronal maturation in organotypic slices (Avossa et al 2003).

-Longitudinal live imaging data could be added to address long-term viability/health of the transduced OLs.

Response to comment 13: We find this suggestion particularly helpful, and we thank the Reviewers for raising this point. Indeed, implementing a longitudinal imaging has strengthened our findings, not only in respect to cell viability (see response to comment 1 and 12). As suggested, we have performed a new series of experiments to examine the expression of AAV-mbp:jGCaMP8s at various time points (DIV14, DIV16 and DIV21), monitoring the same field of view over time, to assess long term viability and health of transduced oligodendrocytes. We also monitored Ca2+ dynamics over a week, plus an intermediate time point for a comprehensive longitudinal assessment. These results are illustrated in Figure 4K of the revised MS, described in the results (page 11, lines 359-60) and discussed at page 13, lines 435-40. To provide a more complete picture of the long-term health of transduced oligodendrocytes we included an additional control by employing propidium iodide (PI) staining to compare cell viability in general and in eGFP positive cells. These results are shown in the new Figure 4-2.

Discussion:

-The authors could provide some comments about potential toxicity (or lack thereof) of the Ca probe sensor.

Response to comment 14: We conducted a thorough analysis at both DIV14 and DIV21 assessing cell death with PI, which revealed a comparable cell death between control and infected slices. We have now included this result in the revised MS in Figure 3-2 (DIV14, DIV21), and in the main text (page 10, lines 327-8).

- The authors suggest that Ca2+ events in myelin sheaths can be sustained by both neuronal activity and intrinsic mechanisms but they did not monitor neuronal activity, which could have been an interesting point to address while recording Ca2+ events in oligodendrocytes.

Response to comment 15: We agree with the Reviewers that monitoring neuronal activity is an intriguing aspect to consider while recording Ca2+ events in oligodendrocytes. We have conducted additional experimental series where in vivo electrophysiological recordings of ventral neurons in spinal organotypic cultures were performed simultaneously to calcium imaging of nearby oligodendrocytes. Subsequently, slices were perfused with an extracellular solution containing TTX. Thus, we monitored the interplay between neuronal activity and Ca2+ dynamics in myelinating oligodendrocytes at DIV14 prior and after removal of action potential driven synaptic activity within the spinal ventral area. We confirmed that upon TTX perfusion only residual miniature postsynaptic currents were detected (Medelin et al.; J Physiol 2016), while Ca2+ activity of oligodendrocytes was reduced in terms of amplitude and duration of the events, without changes in their frequency. These results are now described in the main text (page 10, lines 339-40) and illustrated in Figure 3-3.

-Finally, while this may be out of scope for this technical report, effects of increased neuronal activity (bicuculline treatment?) on calcium signaling in OLs could nicely complement TTX data.

Response to comment 16: We thank the Reviewers for this intriguing suggestion to explore the effects of increased neuronal activity, potentially through bicuculline treatment, on Ca2+ signaling in oligodendrocytes to complement the TTX data at DIV14. While we acknowledge the merit of this proposal, we believe that incorporating experiments to investigate the impact of increased neuronal activity would be beyond the intended scope of our current research.
