# Supplementary figures and images for: Exploring Ca2+ Dynamics in Myelinating Oligodendrocytes through rAAV-Mediated jGCaMP8s Expression in Developing Spinal Cord Organ Cultures
Source: eNeuro. 2024 Jun 3;11(6):ENEURO.0540-23.2024. doi: 10.1523/ENEURO.0540-23.2024 (PMC11151195; doi:10.1523/ENEURO.0540-23.2024)

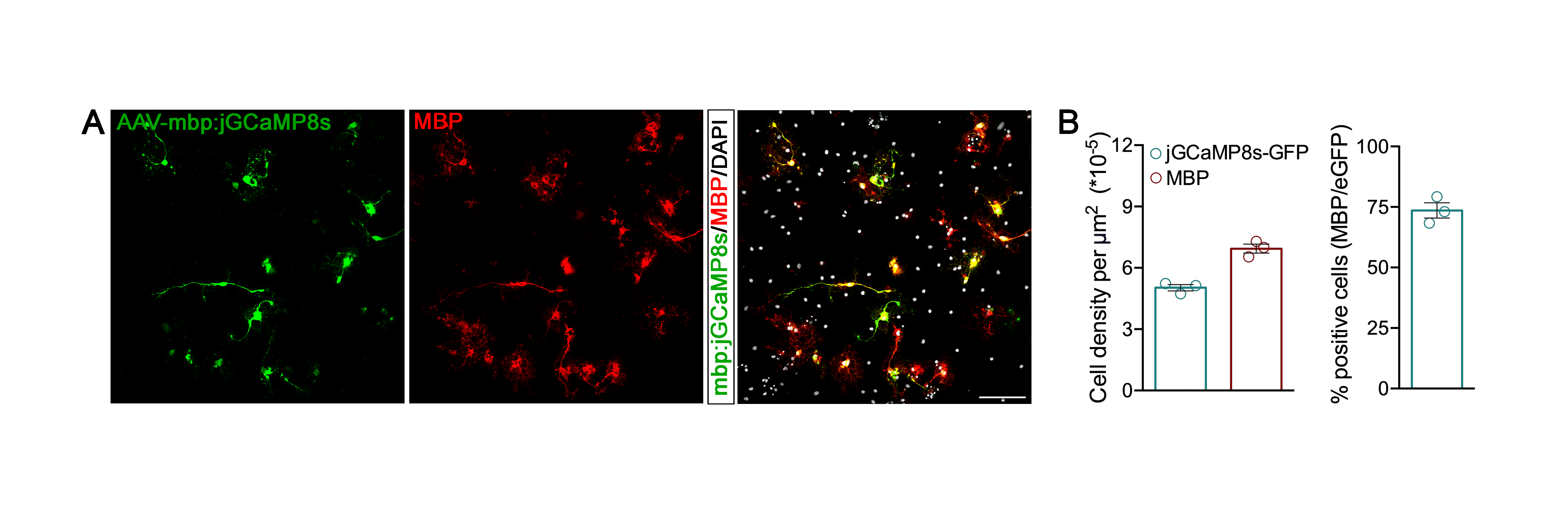

Supplement: Figure 2-1 — A. Representative image of myelinating oligodendrocytes (DIV8) showing eGFP expression for jGCaMP8 s (green) and mature oligodendrocytes (MBP, red). Nuclei (white) were labeled with DAPI dye. Scale bar: 100 μm. B. Quantification of eGFP and MBP cells per µm2 (left panel) and percent of MBP+/eGFP+ cells (right panel). This analysis was performed using maximum intensity projections of 3D z-stack images and quantification was obtained by using the “Analyze particles'‘ function of FIJI ImageJ software; N = 3 oligodendrocytes cultures. Download Figure 2-1, TIF file. [file eneuro-11-ENEURO.0540-23.2024-s003.tif]

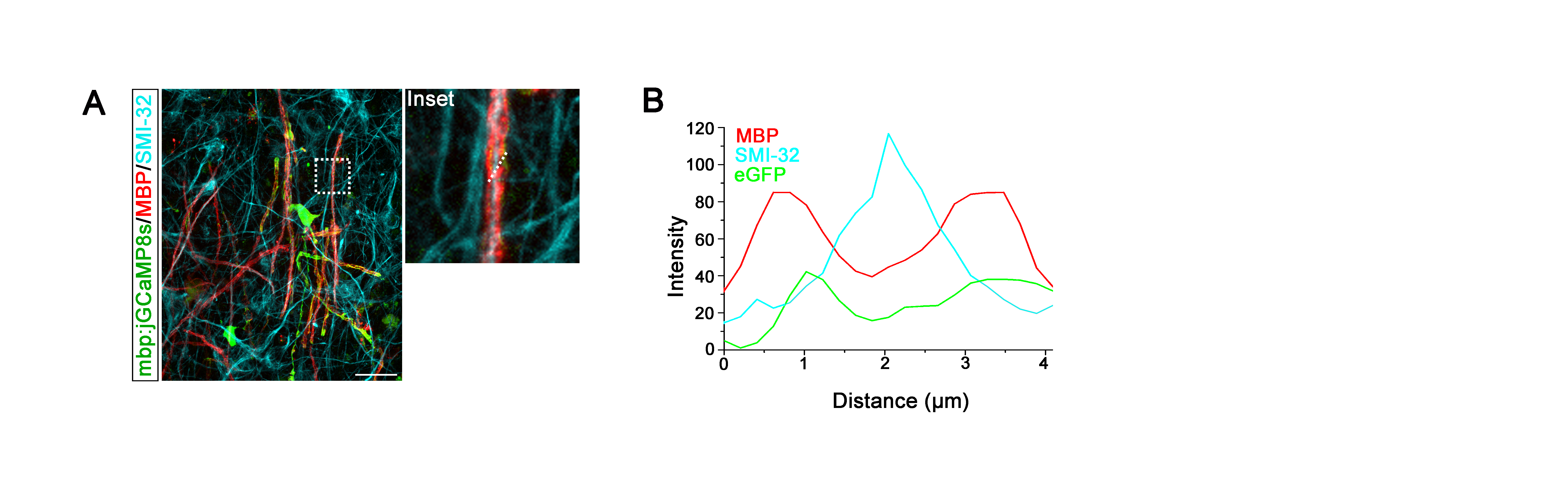

Supplement: Figure 3-1 — A. Representative image of spinal cord organotypic cultures showing AAV-mbp:jGCaMP8 s expression (green), mature oligodendrocytes (MBP, red) and neurons (SMI-32, cyan). Scale bar: 30 μm. Inset represents an image at higher magnification of the region marked in the white box. B. Histogram of MBP, SMI-22 and eGFP (AAV-mbp:jGCaMP8 s) intensity levels along the dashed line indicated in A. Download Figure 3-1, TIF file. [file eneuro-11-ENEURO.0540-23.2024-s004.tif]

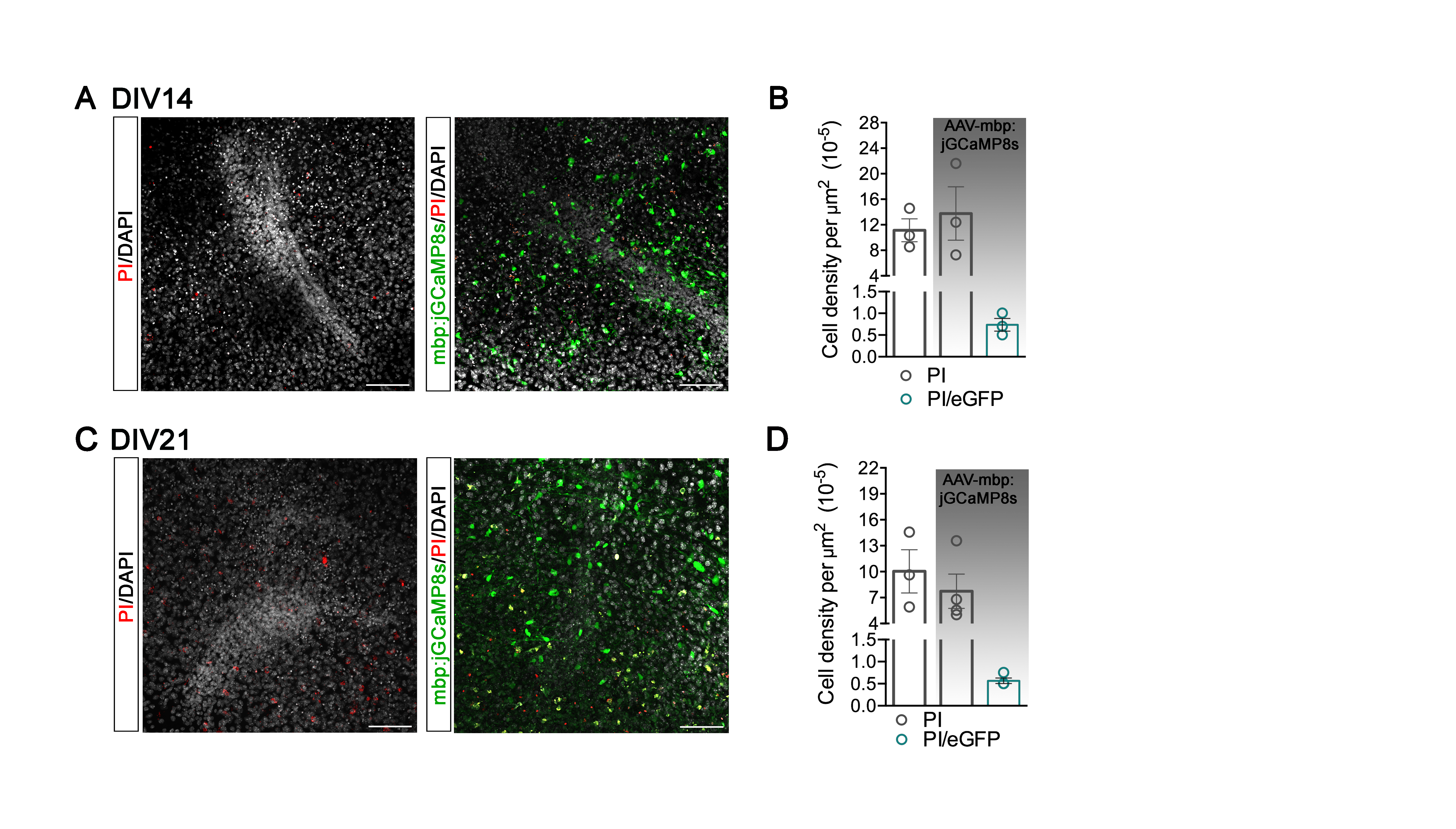

Supplement: Figure 3-2 — A. Representative image of spinal cord organotypic cultures at DIV14 comparing control (left panel) and AAV-mbp:jGCaMP8s-infected samples (right panel, green) with propidium iodide (PI, red). Nuclei (white) were labeled with DAPI dye. Scale bar: 100 μm. B. Quantification of PI and PI/eGFP cell numbers relative to area. C. Representative image of spinal cord organotypic cultures at DIV21 comparing control (left panel) and AAV-mbp:jGCaMP8s-infected samples (right panel, green) with PI (red). Nuclei (white) were labeled with DAPI dye. Scale bar: 100 μm. D. Quantification of PI and PI/eGFP cell numbers relative to area. The number of PI or PI/eGFP-labeled cells was determined using the “Analyze particles'‘ function of FIJI ImageJ software. Control, N = 3; AAV-mbp:jGCaMP8 s, N = 3 spinal cord organotypic cultures. Download Figure 3-2, TIF file. [file eneuro-11-ENEURO.0540-23.2024-s005.tif]

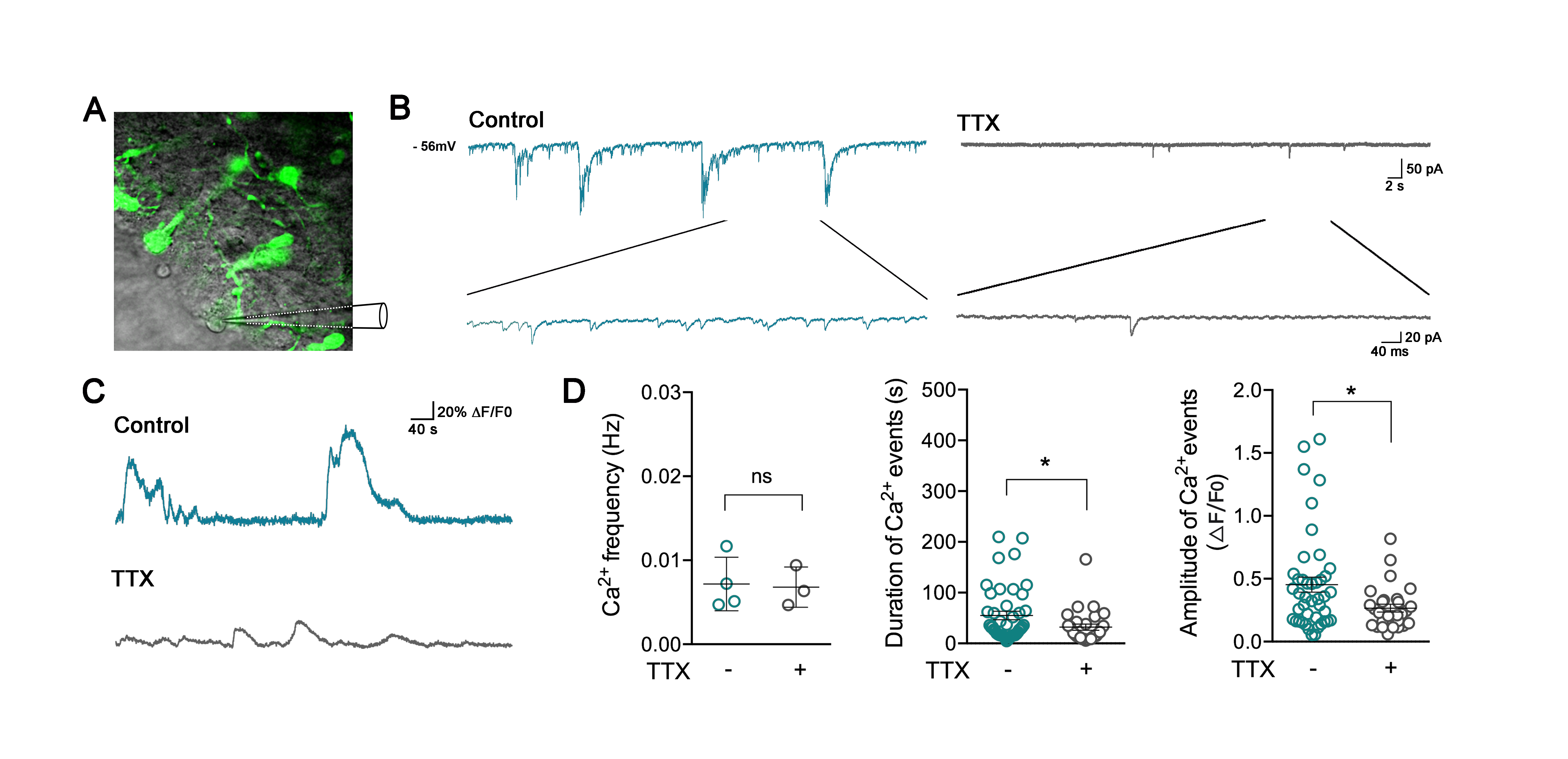

Supplement: Figure 3-3 — A. Maximum intensity projection illustrates a simultaneous neuron whole-cell recording and jGCaMP8s-expressing myelinating oligodendrocytes (green). B. Representative traces of spontaneous neuronal activity recorded from control (green) and upon TTX treatment (gray); insets show abolishment of both individual and clustered spontaneous post-synaptic currents by TTX, with only miniature post-synaptic currents left. C. Representative traces of oligodendrocytes Ca2+ activity expressed as ΔF/F0 over time recorded simultaneously with neuronal activity. D. Distribution of average Ca2+ wave frequency, duration and amplitude of 31 [Control] and 27 [TTX] oligodendrocyte processes, analyzed from N = 4 [Control] and N = 3 [TTX] movies. Two-tailed statistical Mann–Whitney test was used; *P < 0.05; ns, not significant. Download Figure 3-3, TIF file. [file eneuro-11-ENEURO.0540-23.2024-s006.tif]

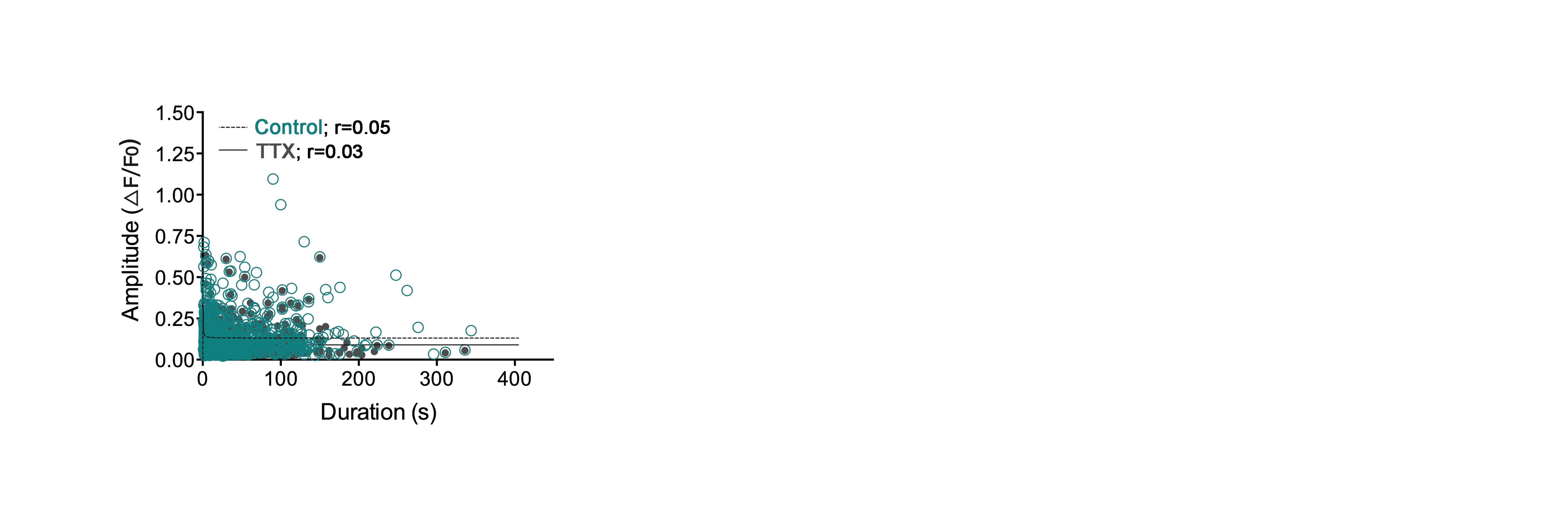

Supplement: Figure 3-4 — Correlation between amplitude and duration of individual Ca2+ events in organotypic spinal cord cultures at DIV14 from 220 [Control] and 135 [TTX] oligodendrocyte processes, analyzed from N = 16 [Control] and N = 10 [TTX] movies. Pearson’s correlation coefficient was calculated using the “Coloc 2'‘ plug-in of FIJI ImageJ software. Two-tailed statistical unpaired t-test was used; r = 0.05, P < 0.13 [Control]; r = 0.03, P < 0.36) [TTX]. Download Figure 3-4, TIF file. [file eneuro-11-ENEURO.0540-23.2024-s007.tif]

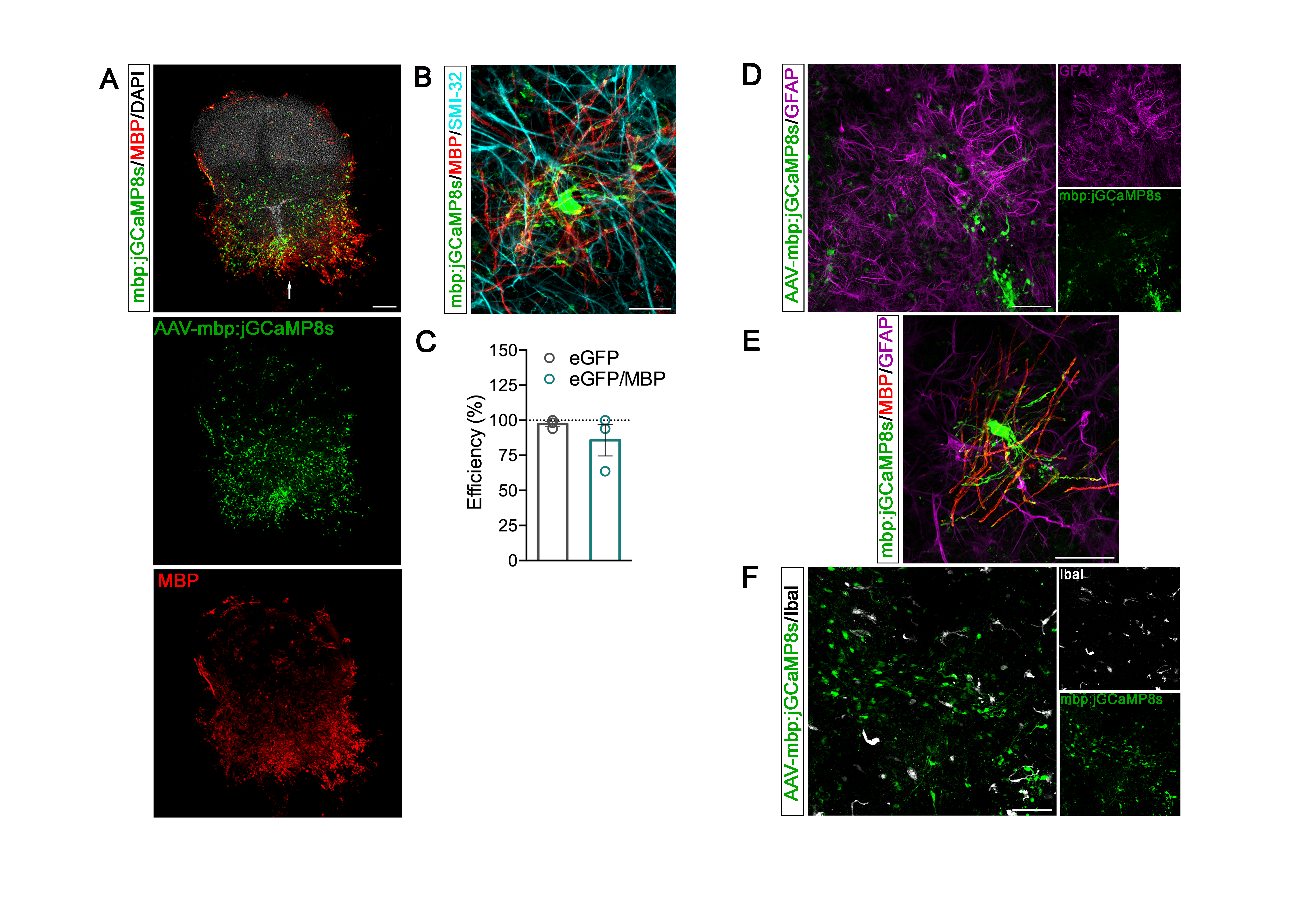

Supplement: Figure 4-1 — A. Representative image of the whole spinal organotypic culture at DIV21 showing AAV-mbp:jGCaMP8 s expression (green) and mature oligodendrocytes (MBP, red). Nuclei (white) were labeled with DAPI dye. Note the ventral region highlighted by the ventral fissure (arrow). Scale bar: 300 μm. B. Representative higher magnification image of the spinal ventral region (same as in A) showing AAV-mbp:jGCaMP8 s expression (green), mature oligodendrocytes (MBP, red) and neurons (SMI-32, cyan). Scale bar: 30 μm. C. Quantification of the AAV-mbp:jGCaMP8 s efficiency in spinal cord organotypic cultures at DIV21. Data are represented as percentages. The analysis was performed using maximum intensity projections of 3D z-stack images, and quantification was obtained by using the “Analyze particles'‘ function of FIJI ImageJ software; N = 3 spinal cord organotypic cultures. D. Representative image of the ventral region of spinal cord showing AAV-mbp:jGCaMP8 s expression (green) and GFAP+ astrocytes (magenta). Scale bar: 100 μm. E. Representative higher magnification image of spinal cord showing AAV-mbp:jGCaMP8 s expression (green), mature oligodendrocytes (MBP, red) and GFAP+ astrocytes (magenta). Scale bar: 30 μm. F. Representative image of ventral region of spinal cord showing AAV-mbp:jGCaMP8 s expression (green) and microglia (IbaI, white). Scale bar: 100 μm. Download Figure 4-1, TIF file. [file eneuro-11-ENEURO.0540-23.2024-s008.tif]

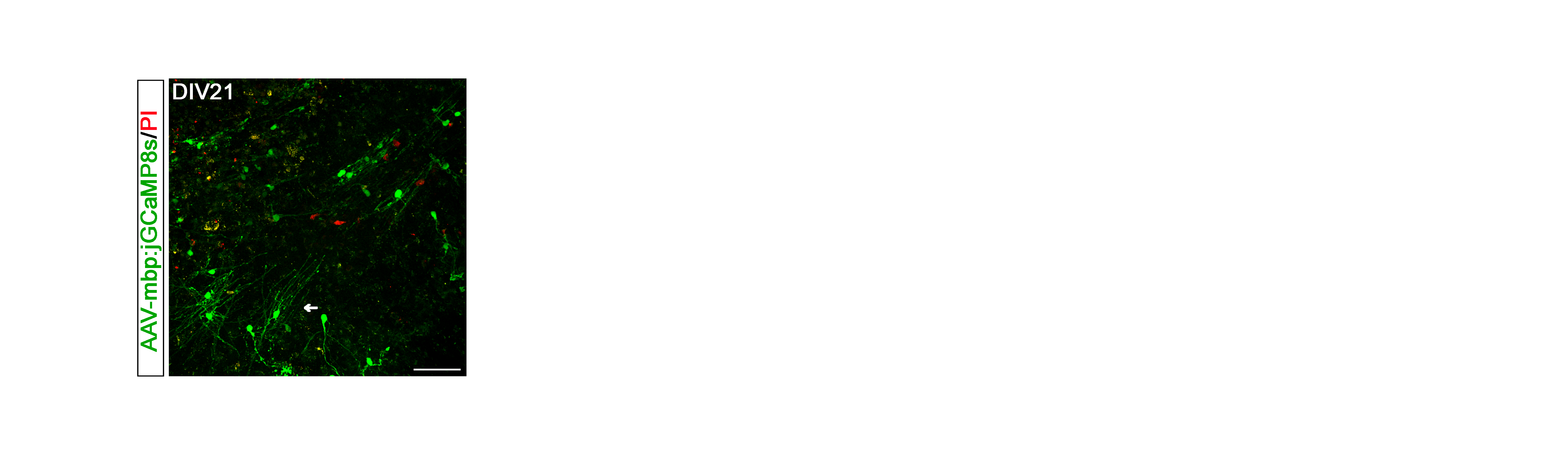

Supplement: Figure 4-2 — Representative image of spinal cord organotypic cultures showing AAV-mbp:jGCaMP8 s expression (green) and PI (red). The arrow indicates the cell that was recorded in the longitudinal in vivo study represented in Figure 4 K. Scale bar: 100 μm. Download Figure 4-2, TIF file. [file eneuro-11-ENEURO.0540-23.2024-s009.tif]
